# Genetic dissection of seedling root architecture under aluminium toxicity in tropical maize (*Zea mays* L.)

**DOI:** 10.3389/fpls.2025.1722162

**Published:** 2026-02-10

**Authors:** Arun M. Channapur, Santosh Kumar, Bhupender Kumar, Preeti Singh, Krishnan P. Abhijith, Akash A., Rakhi Singh, Pooja Belwal, Vijay Prakash, Sandeep Jaiswal, Omkar Maharudra Limbalkar, Saheb Pal, Yamini Thakur, Ganapati Mukri, Yathish K.R., Sanat Kumar Mahanta

**Affiliations:** 1 ICAR-Indian Agricultural Research Institute, Jharkhand, India; 2 ICAR-Indian Institute of Maize Research, Ludhiana, India; 3 ICAR-Indian Agricultural Research Institute, Assam, India; 4 ICAR-Research Complex for North Eastern Hill Region, Meghalaya, India; 5 ICAR-Indian Institute of Agricultural Biotechnology, Ranchi, India; 6 ICAR-Indian Agricultural Research Institute, New Delhi, India; 7 Winter Nursery Center (ICAR-Indian Institute of Maize Research), Hyderabad, India

**Keywords:** aluminum toxicity, candidate genes, GWAS, hydroponics, QTL hotspots, root architectural traits, tropical maize

## Abstract

Aluminum (Al) toxicity is a major production constraint to tropical maize in acidic soils, primarily impairing root growth. This study aimed to dissect the genetic basis of seedling-stage root architectural traits conferring Al tolerance in tropical maize using genome-wide association study (GWAS). A hydroponic protocol was standardized by evaluating seven inbred lines under different Al concentrations, and 300μM AlCl_3_ at day 11 was found optimal for phenotyping. Significant reductions in root length, surface area, volume and tips ranged 24-38%, while diameter increased by 13% under stress in 250 diverse maize inbred lines. Principal component and correlation analyses indicated strong association of root elongation and branching traits, while thickening was largely independent. Genotyping with 60,227 SNPs revealed three subpopulations with moderate linkage disequilibrium decay (65.4 kb), supporting high-resolution GWAS. Mixed Linear Model analysis detected 44 significant SNPs across eight chromosomes, explaining 9.68-18.43% of phenotypic variance. Eight QTL clusters were identified, including two major hotspots on chromosomes 8 and 3 designated as “QTL hot spot A” and “QTL hot spot B” with seven and five major QTLs respectively. Candidate gene analysis highlighted 33 functionally relevant genes linked to stress tolerance, including glutathione S-transferases, Dehydroascorbate reductases, MATE transporters, and regulators of STOP1 stability and activity. In-Silico expression analysis confirmed stress-responsive regulation of several promising genes. Collectively, this study provides genomic regions and candidate genes underpinning root-based Al tolerance, offering valuable targets for marker-assisted breeding and genomic prediction in tropical maize.

## 1 Introduction

Maize (*Zea mays* L.) is one of the most commercially significant cereal crops, serving as a crucial source of food, feed, fodder, ethanol, oil and industrial raw materials worldwide. It is cultivated on nearly 205 million hectares globally, producing nearly 1263 million tons of grain and contributing substantially to food security in Asia, Africa and Latin America (Anonymous, 2025). In recent years, the rising global demand for maize has necessitated the urgent need to enhance its productivity. However, this requirement is constrained by diminishing land and water resources, coupled with increasing biotic and abiotic stresses. Among abiotic stresses, aluminum toxicity in acidic soils is a major constraint to maize production. Under low soil pH conditions, the insoluble forms of aluminum are solubilized, resulting in the release of phytotoxic Al³^+^ ions, primarily existing as the hexaaqua complex Al(H_2_O)_6_^3+^. This transformation distinguishes the total aluminum content of the soil from its biologically active and toxic fraction, which severely impairs root growth and nutrient uptake in sensitive genotypes. Approximately 40-50% of the world’s potentially arable land is affected by soil acidity, which covers about 3950 million hectares of the world’s ice-free land (Eswaran et al., 1997). In India alone, out of 157 million hectares of cultivable land, nearly 19.75% (31 Mha) is categorized as acidic (Majji et al., 2012). Grain yield reductions up to 71% have been reported under acidic conditions, primarily due to soil acidity-induced aluminum toxicity (SAIAT) (Tandzi et al., 2015). Developing aluminum tolerant maize varieties enables better root growth, nutrient uptake and overall plant performance under acidic soil conditions. Such improvement not only enhances productivity in stress prone environments but also allows the effective utilization of vast acidic land resources, thereby supporting food security and sustainable agricultural development.

Conventional ameliorative practices such as liming have limited effectiveness in mitigating acidity in sub-surface soils (Pal et al., 2014). Consequently, the development of acid-soil-tolerant maize genotypes represents an economically viable, eco-friendly, and sustainable approach to enhance productivity on such marginal soils. In maize (*Zea mays* L.), aluminum toxicity primarily manifests as inhibited root growth, reduced root elongation, and altered root morphology, which impair water and nutrient absorption leading to stunted shoot growth and overall biomass reduction. The toxicity symptoms include shortened and thickened roots, decreased root surface area and leaf chlorosis under high Al exposure (Zhou et al., 2009; Zhang et al., 2021).

Tolerance to Al toxicity varies widely both within and between the species (Caniato et al., 2007; Famoso et al., 2010). Additionally, genetic variation for aluminum tolerance exists within maize, with tolerant genotypes exhibiting higher organic acid exudation, particularly citrate, which chelates Al ions and alleviates toxicity in the rhizosphere. Such inherent variability suggests the presence of a genetic basis for Al toxicity tolerance, thereby offering opportunities for targeted improvement through modern breeding strategies. With the advancement of molecular tools, Genome-Wide Association Studies (GWAS) have emerged as an effective alternative to conventional QTL mapping, allowing genome-wide scanning for variants linked to complex traits. Unlike biparental mapping, GWAS provides greater resolution and captures broader allelic variation by utilizing natural diversity present in diverse germplasm, thereby facilitating the precise identification of genomic regions controlling quantitative traits. Given the polygenic nature of Al tolerance (Guimarães et al., 2010), GWAS has already been successfully applied in wheat (Navakode et al., 2014), rice (Famoso et al., 2011; Tao et al., 2018), common bean (Ambachew and Blair, 2021), and rapeseed (Gao et al., 2020; Zhou et al., 2022) to dissect the Al tolerance, but the genetic information available in maize remains limited. While the earlier studies in maize, using traditional bi-parental mapping approaches have reported a total of 14 QTLs associated with Al stress tolerance distributed across chromosomes *viz*., 2, 4, 5, 6, 7, 8, 9 and 10 (Sibov et al., 1999; Ninamango-Cárdenas et al., 2003; Conceição et al., 2009; Coelho et al., 2019), although limited studies have systematically dissected the genetic basis of Al toxicity tolerance in maize, particularly using large and diverse tropical germplasm, this study addresses this gap by analyzing a large tropical panel under controlled Al stress with high-density SNP-based GWAS.

Hence, in the present study, three experiments were conducted to dissect the genetic basis of Al toxicity tolerance in maize. First experiment represented a preliminary screening of the seven maize accessions under the different Al concentrations to establish a suitable experimental system and to find the most suitable concentration to induce differential stress responses in maize. The second experiment was carried out to phenotype 250 maize accessions under the Al stress conditions, based on the root architectural traits (total root length, root surface area, average root diameter, root volume, and number of root tips). Finally, GWAS was done to identify QTLs for Al toxicity and to analyze candidate genes within the detected QTL regions.

## 2 Materials and methods

### 2.1 Growth conditions

All experiments were carried out in controlled conditions at ICAR-IARI, Jharkhand, India. Seeds were germinated for three days in the dark at 25° C and 95% RH in a seed germinator by standard paper towel method (International Seed Testing Association (ISTA), 2024) where seeds were placed in between the two moistened germination papers (40 × 33 cm). On the fourth day, uniform seedlings were transferred to hydroponic containers (40 × 52 × 11 cm) in a temperature controlled room maintained at 28 ± 1°C during the day and 25 ± 1°C at night, with a 14 h light/10 h dark photoperiod and relative humidity of approximately 80–90%. Each container had a perforated lid for accommodating the seedlings and PVC tubes were installed at the bottom of the lid to separate the roots of the individual seedlings. The containers were filled with 16 liters of modified Magnavaca-II medium (Jaiswal et al., 2022) having the following composition: KCl (1.045 mM), NH_4_H_2_PO_4_ (45 µM), NH_4_Cl (1.45 mM), CaCl_2_·2H_2_O (405 µM), Ca (NO_3_)_2_.4H_2_O (595 µM), MgSO_4_·7H_2_O (200 µM), Mg (NO_3_)_2_.6H_2_O (655 µM), MnCl_2_·4H_2_O (11.8 µM), H_3_BO_3_ (33 µM), ZnSO_4_·7H_2_O(3.06 µM), CuSO_4_.5H_2_O (0.8 µM), Na_2_MoO_4_·2H_2_O (1.07 µM), and Fe-HEDTA (77 µM) with different AlCl_3_ concentrations ranging from 200 μM to 1500 μM. The nutrient solution was continuously aerated and was replaced once every week throughout the experiment. The pH of the medium was monitored daily and maintained at 4.2 by adjusting with HCl and NaOH.

### 2.2 Protocol standardization for hydroponic aluminum stress screening

Seven elite maize inbred lines (LM-13, BML-7, BML-45, CML-425, PML-5, PML-9 and DQL-2070) obtained from the ICAR-IIMR, Ludhiana, India, were used in the preliminary experiment to standardize hydroponic aluminum stress screening conditions. For each inbred, two replications were maintained with three seedlings per replication and the seedlings were subsequently subjected to six different concentrations of the AlCl_3_ (200 μM, 300 μM, 600 μM, 900 μM, 1200 μM and 1500 μM) apart from the control (0 μM) under the conditions described above. The data were recorded from second day after transfer to the hydroponic medium and was continued daily up to 7 days. Different root architectural traits *viz.* total root length (TRL, cm), root surface area (RSA, cm²), average root diameter (AD, mm), root volume (RV, cm³) and number of root tips (NRT) were observed to identify the most suitable concentration and exposure period for subsequent phenotyping the association mapping panel. For scanning, roots were gently washed with distilled water and spread in a single, non-overlapping layer in a transparent tray filled with a thin layer of water to avoid shadowing. Root images were acquired using the Epson Expression 13000XL flatbed scanner at a resolution of 600 dpi. Image analysis was performed using *WinRHIZOPro* with the default grey-scale threshold value of 128 for root background separation. All samples were processed using identical scanning and analysis parameters to ensure consistency and reproducibility.

### 2.3 Phenotyping of the association mapping panel

Following the standardization experiment, a diverse panel of 250 tropical maize inbred lines, sourced from ICAR-IIMR, Ludhiana, India, was subjected to phenotyping (**Supplementary Table S1**). The panel comprised normal field corn, quality protein maize, popcorn and sweet corn, exhibiting diversity in kernel color (yellow, white, orange and dark orange), maturity groups (early, medium and late), plant height (short to tall) and ear placement (low to high) (Kumar et al., 2022). For each inbred, two replications were maintained with three seedlings per replication and the seedlings were subsequently subjected to aluminum stress using 300 µM AlCl_3_(as standardized in the preliminary experiment) apart from the control (0 μM) under the conditions described above. Uniform growth stage across accessions was ensured by selecting only evenly germinated seedlings at the same developmental stage for transfer to the hydroponic system. The data were recorded on eleven-day old seedlings (eight days after the transfer to the hydroponic medium as standardized preliminary experiment) for different root architectural traits as was observed in the protocol standardization experiment. For each replication, data were recorded on three individual seedlings, and the mean value was used for statistical analysis.

### 2.4 Genotyping

Genotypic data for the association mapping panel (AMP), consisting of 250 tropical maize inbred lines, were obtained using a genotyping-by-sequencing (GBS) approach on the Illumina HiSeq platform. Genomic DNA was extracted from fresh leaf tissue of five plants per line using the GenElute Plant Genomic DNA Miniprep Kit. DNA concentrations were quantified using a Qubit fluorometer and normalized for library preparation. Libraries were prepared by digesting genomic DNA with the *ApeKI* restriction enzyme, followed by ligation with barcoded adapters, and sequencing was carried out on the Illumina HiSeq platform using TruSeq paired-end technology (Kumar et al., 2022). The sequencing generated FASTQ files, were quality-filtered to remove low-quality reads and barcode errors. Reads were aligned to the maize reference genome B73 (Zm-B73-REFERENCE-NAM5.0) using the Burrows-Wheeler Alignment (BWA) tool. Variant calling was performed using FreeBayes, and duplicate removal was done with PICARD. SNPs were filtered using VCFtools, retaining biallelic SNPs with minor allele frequency (MAF) above 0.05 and a maximum of 20% missing data, resulting in a final set of 60,227 high-quality, polymorphic SNPs distributed across all 10 maize chromosomes, providing good genome-wide coverage (Kumar et al., 2022). The genotypic data is available with ICAR-IIMR, Ludhiana, India, which has been used to do the GWAS study.

### 2.5 Population structure and principal component analysis

Population structure was assessed using STRUCTURE V.2.3.4 software and principal component analysis (PCA) in TASSEL V.5.2. STRUCTURE analysis suggested the presence of three possible subpopulations within the AMP. PCA further characterized genetic variation and kinship analysis in TASSEL estimated relatedness among lines. These analyses confirmed overall low relatedness among most lines but clear clustering of some related lines, supporting the need to account for structure in association analyses. Covariates derived from STRUCTURE and PCA were included in GWAS models to control confounding effects due to population stratification and relatedness (Kumar et al., 2022).

### 2.6 Linkage disequilibrium estimation

Linkage disequilibrium (LD) analysis was performed using SNP datasets filtered by minor allele frequency (MAF) thresholds of ≥0.05, ≥0.10, and ≥0.20. A total of 60,227 SNPs meeting the MAF ≥0.05 criterion was used in the primary dataset. LD was estimated as the squared allele-frequency correlation (r²) between all pairwise SNPs across the genome. LD values were calculated across inter-marker distance intervals ranging from < 0.0001 Mb to 100 Mb within each chromosome. LD decay was assessed on a chromosome-wise basis, wherein a LOESS smoothing curve was fitted to the R²–distance relationship for each chromosome, and the decay distance was defined as the point at which the smoothed R² value dropped below 0.2. The analysis of haplotype block distribution was conducted using TASSEL V.5.2, while LD decay visualization was carried out in RStudio (4.4.3) using the tidyverse, ggplot2, and dplyr with a LOESS span of 0.1 and 95% confidence intervals enabled (se = TRUE).

### 2.7 Genome-wide association analysis

Genome-wide association analysis (GWAS) was conducted in TASSEL V.5.2 using both General Linear Model (GLM) and Mixed Linear Model (MLM) frameworks, with the MLM primarily employed in this study to account for population structure and kinship. Phenotypic data on five root architectural traits collected under control and aluminum stress conditions through hydroponic screening, however, only data from the aluminum stress treatment (as per standardized concentration and duration) were used for association mapping and subsequent candidate gene identification. Significant marker-trait associations were identified using the MLM approach, which incorporated principal component and kinship covariates to effectively control for population structure and relatedness among genotypes. The resulting associations were visualized using Manhattan plots and significant SNPs were selected based on stringent log_10_ (p)>5.0. A –log_10_(p) > 5 threshold was adopted and verified that the major associations remained consistent under Bonferroni corrections.

### 2.8 Candidate gene identification

The candidate genes were searched against the maize reference genome B73 (Zm-B73-REFERENCE-NAM5.0). The average LD decay rate estimated in this population is approximately 65.4 kb. The same window of 65.4 kb was applied on either side of the significant SNP to investigate the local LD pattern and search for putative candidate genes. Gene models within the candidate regions were obtained from the Maize Genetics and Genomics Database (MaizeGDB) (https://www.maizegdb.org). Promising candidate genes were classified based on the gene annotation and further considered for discussion for their potential role in aluminum stress.

### 2.9 In silico expression and co-expression analyses of candidate genes

To reveal the stress-responsive expression pattern of selected candidate genes, the whole genome transcriptome data sets of abiotic stresses were collected for drought (NCBI Bio- projects: PRJNA782891; PRJNA545969), heat (NCBI Bio-project: PRJNA506720), salinity (NCBI Bio-project: PRJNA527733) and nitrogen starvation (NCBI Bio-project: PRJNA436973). The expression values of candidate genes were retrieved from corresponding expressions datasets and calculated log 2 FC values for uniform representation.

### 2.10 Statistical analysis and data visualization of phenotypic data

Descriptive statistics including mean, standard deviation (SD), co-efficient of variation (CV%), minimum and maximum were computed for each trait using the dplyr and tidyr packages in RStudio (version 4.4.3). Analysis of variance (ANOVA) was performed under a Completely Randomized Design (CRD) using appropriate packages in R to partition genotypic and error variances. Statistical analysis for the standardization study was performed using a three-way factorial Analysis of Variance (ANOVA) to evaluate the effects of Genotype (7 levels), Aluminum Concentration (7 levels) and Duration (7 levels) on root traits. The mathematical model included main effects for all three factors, as well as all two-way and three-way interactions.


$$Y_{ijkl}=\mu +\alpha_{i}+\beta_{j}+\gamma_{k}+{\left(\alpha \beta \right)}_{ij}+{\left(\alpha \gamma \right)}_{ik}+{\left(\beta \gamma \right)}_{jk}+{\left(\alpha \beta \gamma \right)}_{ijk}+\epsilon_{ijkl}$$

*Y_ijkl:_* The observed trait value*; μ:* The overall population mean*; α_i_:* The main effect of Genotype, *β_j_*: The main effect of Aluminum Concentration; *γ_k_*: The main effect of Duration; *(αβ)_ij_, (αγ)_ik_, (βγ)_jk_, (αβγ)_ijk_:* Interaction Terms*; ϵ_ijkl_:* The random residual error.

Phenotypic-based PCA was performed using the R package “FactoMineR version 2.4”. Graphical representation of PCA results was done with R package “factoextra version 1.0.7” (Kassambara and Mundt, 2020). Pearsons correlation coefficient among the root architectural traits was calculated and graphically represented using an R package “corrplot” (Wei and Simko, 2021).To compare control and treatments, boxplots with jittered genotype points were constructed and statistical differences were assessed using the non-parametric wilcoxon rank-sum test implemented in ggpubr (Kassambara, 2018), with significance levels denoted by asterisks (p< 0.05, p< 0.01, p< 0.001, p< 0.0001).

## 3 Results

### 3.1 Protocol standardization for hydroponic aluminum stress screening

To establish a suitable screening system for phenotyping and for the subsequent GWAS analysis, seven different maize inbred lines were tested under seven different aluminum concentrations. Increase in Al concentration markedly reduced total root length (TRL), root volume (RV), root surface area (RSA), and number of root tips (NRT), while slight increase in average root diameter (AD) was observed. The ANOVA revealed highly significant effects (p < 0.001) of genotype, Al concentration and exposure duration across all traits (**Table 1**). Among the seven aluminum concentrations, at ≥600 μM, root growth was severely inhibited in all genotypes, thereby reducing the ability to discriminate tolerance levels. In contrast, 300 μM AlCl_3_ concentration on day 11 (8 days after transfer) showed the higher genotypic variation for all the root traits. At this concentration, genotypes such as PML-9, CML-425 and LM-13 maintained higher root trait values compared with genotypes such as BML-45 and DQL-2070, which showed higher reduction. Based on this observed variation, 300 μM AlCl_3_ on day 11 (8 days after transfer) was selected for phenotyping of association mapping panel under the Al stress.

**Table 1:** Effect of aluminum stress on root architectural traits in the protocol standardization experiment used to establish the optimal conditions for phenotyping the GWAS panel.

Sources of variation	Df	RV	TRL	NRT	RSA	AD
Genotype	6	2.02 ***	3935 ***	68766 ***	339 ***	1.574 ***
Al Conc.	6	12.608 ***	88552 ***	1718927 ***	10647 ***	2.743 ***
Day	6	12.322 ***	3921 ***	61444 ***	7394 ***	10.697 ***
Genotype × Al Conc.	36	0.276 ***	1064 ***	22689 ***	230 ***	0.17 ***
Genotype × Day	36	0.09 ***	22	577	37 ***	0.077 **
Al Conc. × Day	36	0.191 ***	233 ***	3672 ***	407 ***	0.481 ***
Genotype × Al Conc. × Day	216	0.033	15	616	29 ***	0.071 ***
Residuals	686	0.032	30	925	7	0.042

TRL, total root length (cm); RSA, root surface area (cm^2^); AD, average root diameter (mm); RV, root volume (cm^3^), and number of root tips (NRT); Significance level, ** = p < 0.01, *** = p < 0.001.

### 3.2 Phenotypic characterization of root architectural traits under Al toxicity conditions

**Figure 1:**
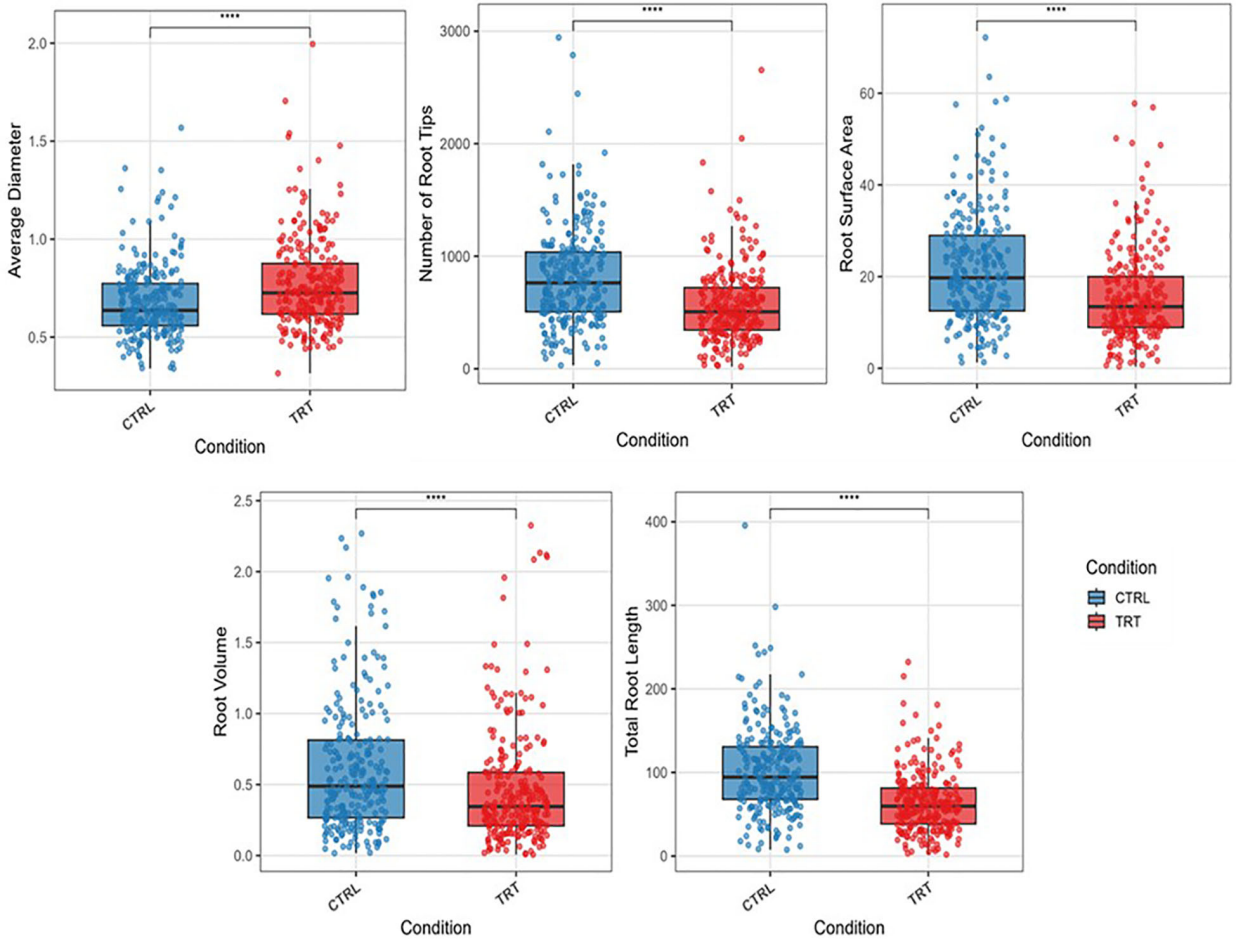
Box plot showing the distribution of traits within the population under control and Al stress conditions.

**Figure 2:**
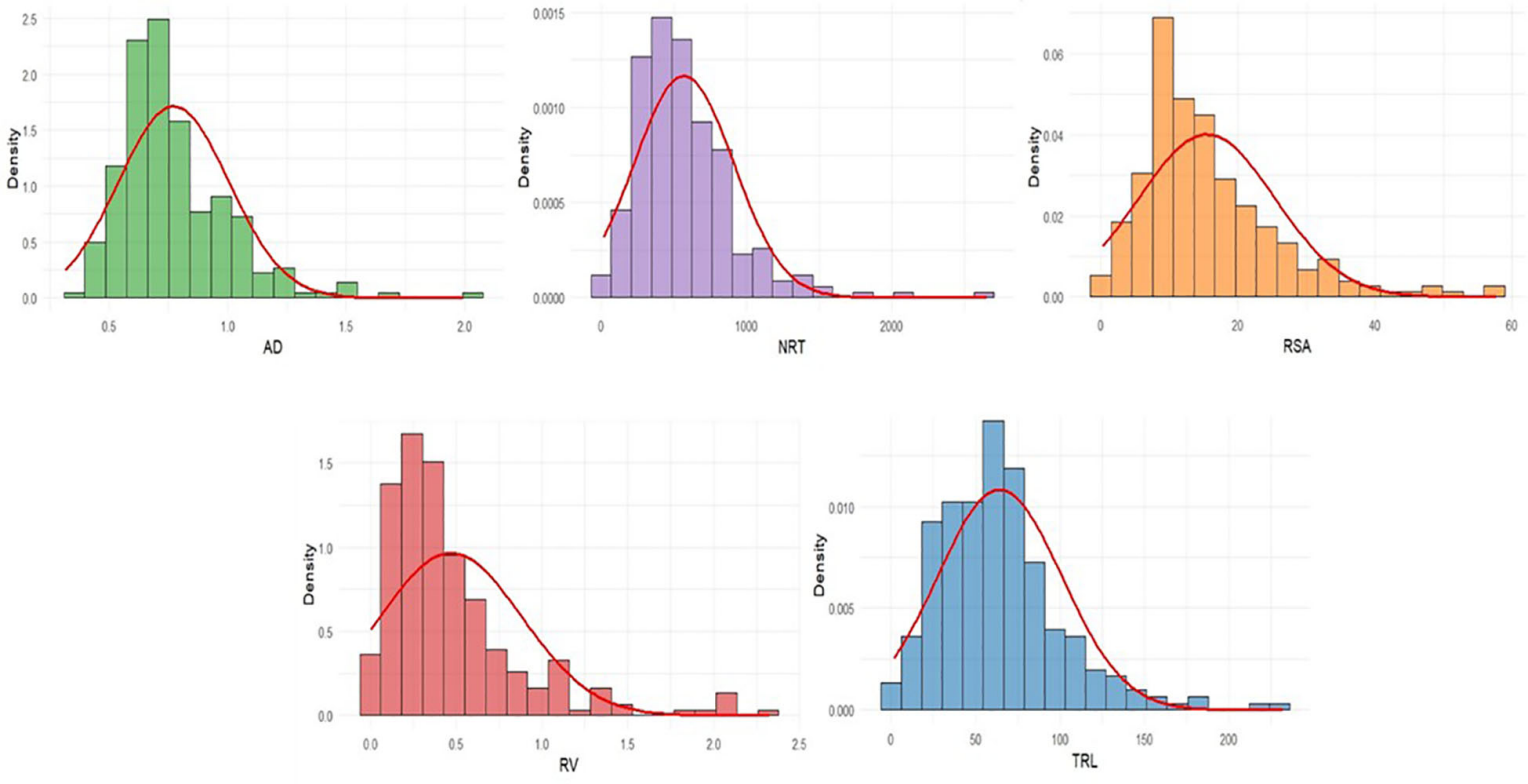
Histogram showing the frequency distribution of root architectural traits under the Al stress conditions.

Our study examined the effects of aluminum stress (300 μM AlCl_3_) on root architectural traits *viz*., TRL, RSA, AD, RV and NRT in 250 maize accessions. All the phenotypic traits showed a statistically significant difference between aluminum stress and control (**Table 2**, **Figure 1**). Total root length (TRL) ranged from 1.75 to 244.6 cm with a mean of 64.39 cm, corresponding to a 38.4% reduction compared to control, while root surface area (RSA) ranged from 0.33 to 60.80 cm² with a mean of 15.49 cm² (35.2% reduction). Root volume (RV) varied between 0.01 and 2.43 cm³ (mean 0.47 cm³; 24.1% reduction), and the number of root tips (NRT) ranged from 18.37 to 2971 with a mean of 570.23 (33.4% reduction). In contrast, average root diameter (AD) increased by 13.2% under stress, spanning from 0.31 to 2.02 mm. The highest variability under stress was observed for AD (CV = 4.95%) and NRT (CV = 4.91%). Overall, the extent of available variation for all the root architectural traits under aluminum stress was suitable for identifying marker trait association for root architectural traits (**Figure 2**).

**Table 2:** Descriptive statistics and ANOVA of root architectural traits under control and Al treated conditions across 250 maize accessions.

Traits	C	T	“T” significance	Range (Control)	Range (Treatment)	CV % (Control)	CV % (Treatment)	“G” significance	GXT	Replication
TRL	104.47	64.39	***	6.49-403.33	1.75-244.6	4.88	4.53	***	***	ns
RSA	23.91	15.49	***	1.6-76.3	0.33-60.80	4.48	4.16	***	***	ns
AD	0.68	0.77	***	0.3-1.91	0.31-2.02	4.66	4.95	***	***	ns
RV	0.62	0.47	***	0.02-2.35	0.01-2.43	4.65	4.25	***	***	ns
NRT	856.65	570.23	***	47-3132	18.37-2971	4.71	4.91	***	***	ns

TRL, total root length (cm); RSA, root surface area (cm^2^); AD, average root diameter (mm); RV, root volume (cm^3^), and number of root tips (NRT); GXT, Genotype x Treatment; CV %, coefficient of variation; C, control; T, AI treatment (300μM AlCl3); ns: non significant; Significance level, *** = p < 0.001.

### 3.3 Principal component analysis and correlation analysis of root traits under Al toxicity

**Figure 3:**
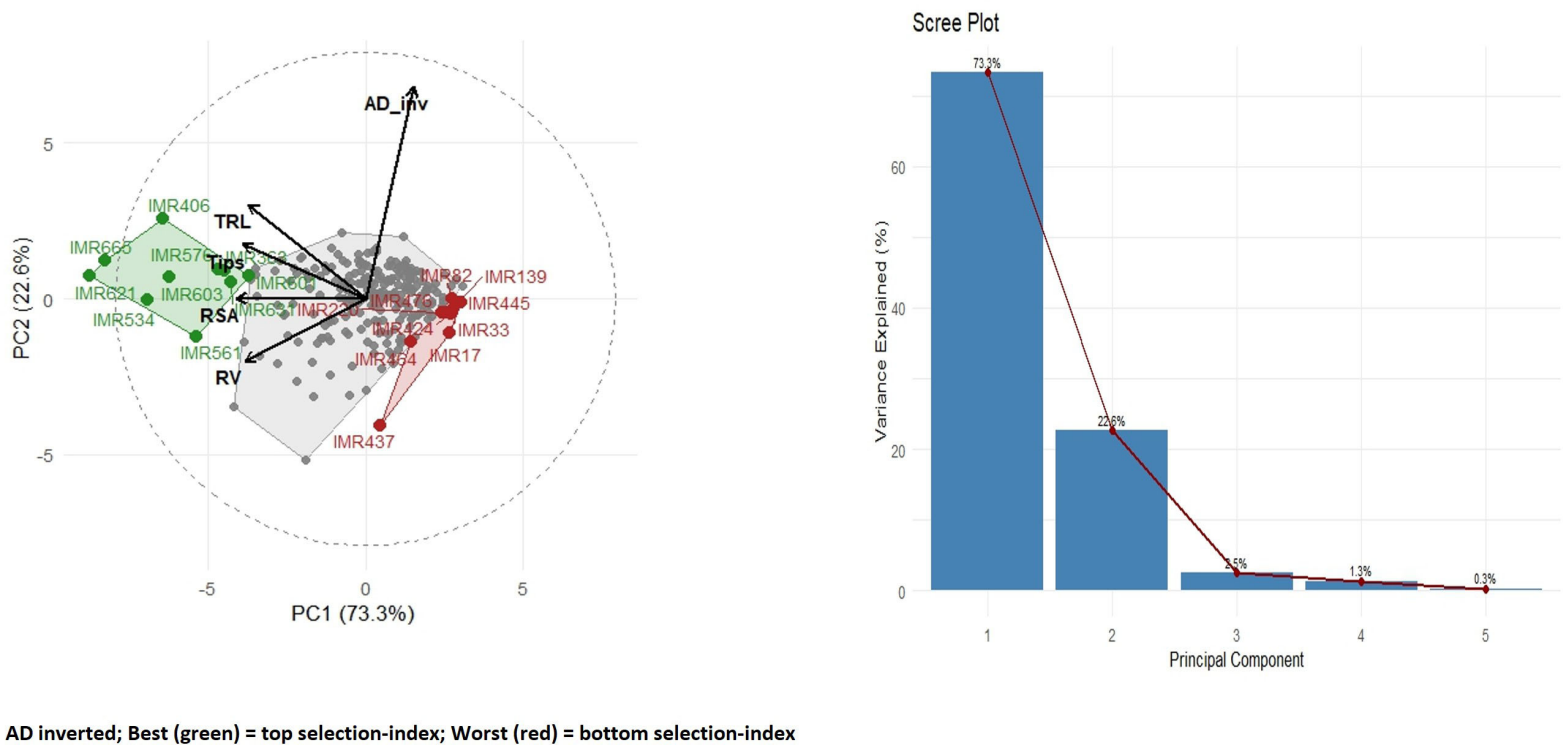
Principal component analysis (PCA) based on different root architectural traits under the Al stress conditions.

The PCA was performed to decipher the population structure of 250 inbred lines with respect to root architectural traits. Using the data for root architectural traits, PCA was carried out which divided the genotypes into multiple clusters. The first two principal components explained 95.9% of the total variance, with PC1 and PC2 accounting for 73.3% and 22.6% of the variation, respectively (**Figure 3**). Based on the loadings of the genotypes, the genotypes were distributed in the factor plane of a scatter plot (**Figure 3**). The genotypes IMR665, IMR406, IMR603 and IMR621 were associated with the higher root architectural trait values under the Al toxicity conditions. Contrastingly, the genotypes IMR33, IMR58, IMR388, IMR175 and IMR493 were associated with the lower root trait values. The selected inter-crossing may facilitate the development of genotypes with greater resistance to Al stress by combining alleles for different root traits.

**Figure 4:**
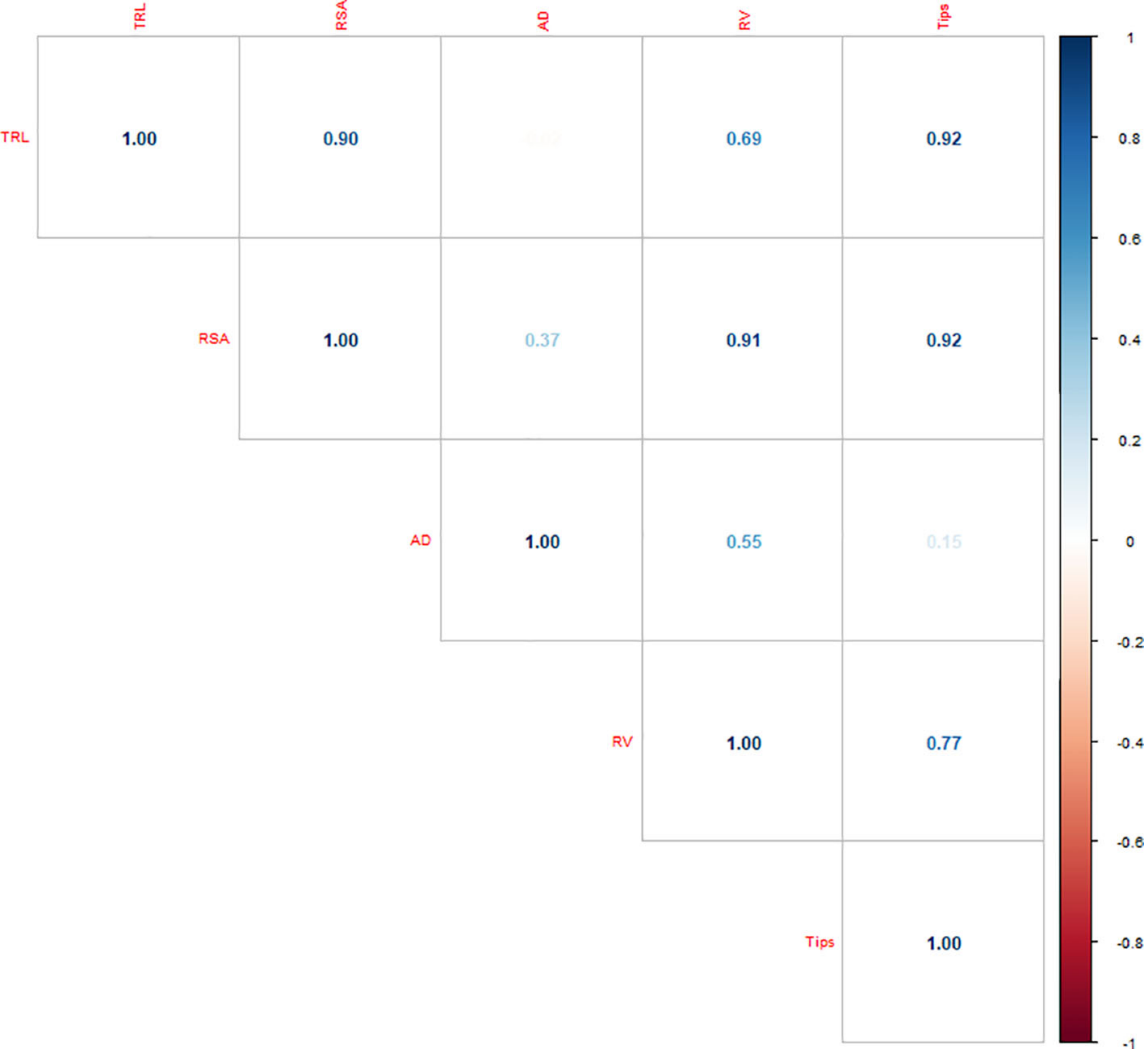
Correlation analysis based on different root architectural traits under the Al stress conditions.

Correlation analysis among root traits showed strong positive associations among most parameters (**Figure 4**). TRL was significantly correlated with RSA (*r* = 0.897), RV (*r* = 0.693), and NRT (*r* = 0.923). RSA also exhibited strong correlations with RV (*r* = 0.909) and NRT (*r* = 0.918), while RV was positively correlated with NRT (*r* = 0.771). In contrast, AD showed very weak associations with TRL (*r* = –0.024) and NRT (*r* = 0.153), and moderate correlations with RSA (*r* = 0.367) and RV (*r* = 0.547). Collectively, TRL, RSA, RV and NRT form a closely related group of traits reflecting root system size and branching, while AD remained largely independent.

### 3.4 Genetic population structure and whole genome LD

**Figure 5:**
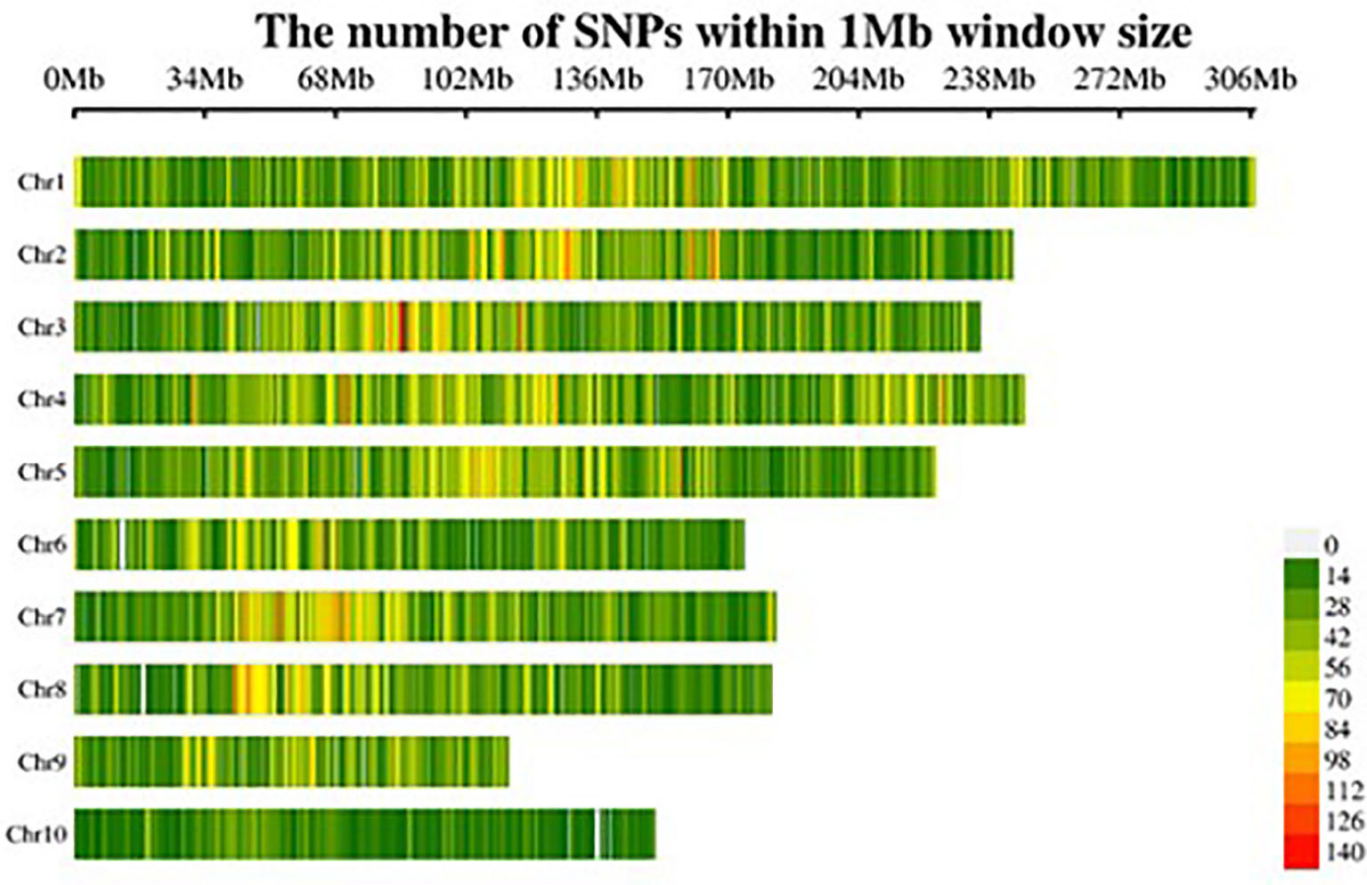
Density of SNPs per chromosome. The horizontal axis displays the chromosome length (Mb), and the vertical axis denotes the chromosome number, with SNP density displayed in various colors.

**Figure 6:**
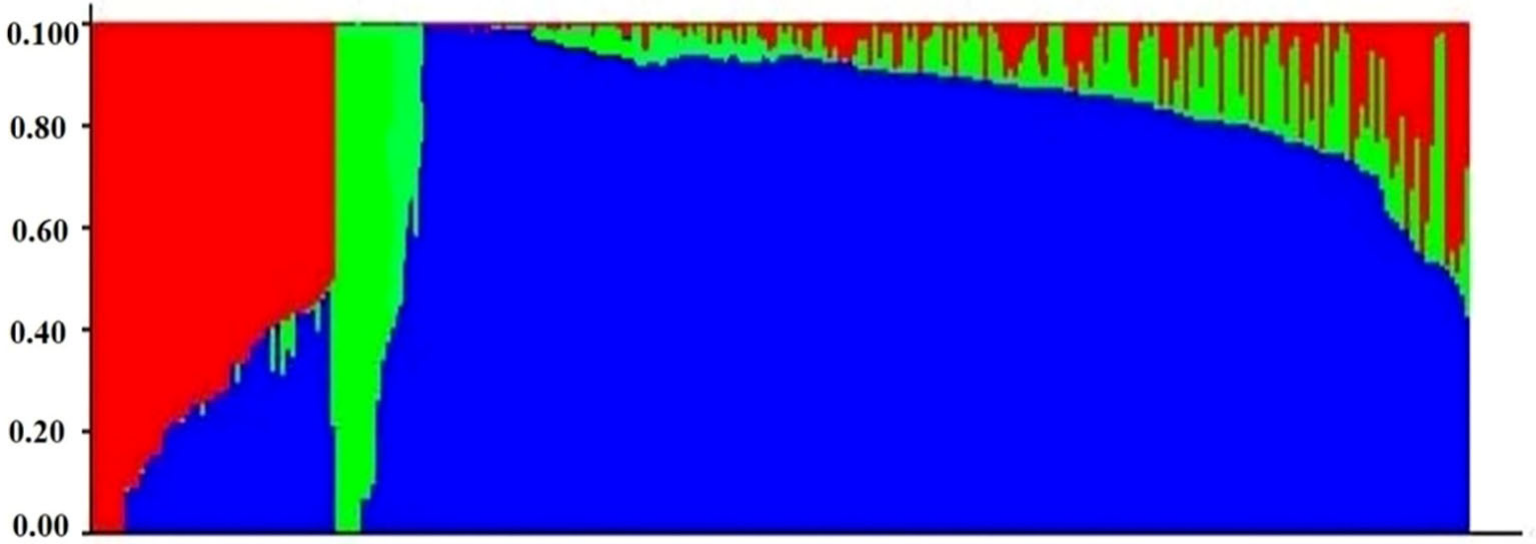
Bayesian-based population structure analysis. Bar plots for K = 3; each genotype is represented by a single vertical line, and each color represents one cluster.

**Figure 7:**
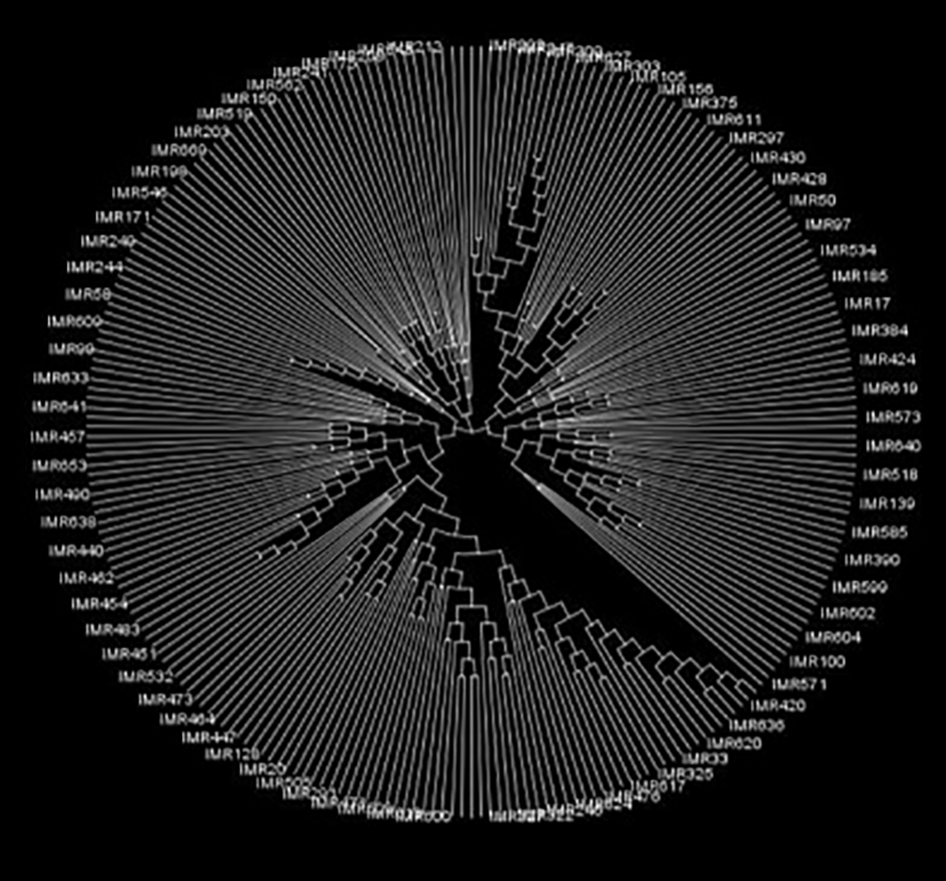
Neighbor-joining tree based on a distance matrix.

To explore the genetic relationships and structure within the 250 inbreds, genotypic data comprising 60,227 high-quality polymorphic SNPs distributed across all 10 chromosomes were analyzed (**Figure 5**). A majority of pair-wise relative kinship values (58.5%) were found to be near-zero or zero, suggesting limited allelic redundancy and a broad genetic base among the inbreds. Only 19.3% of the kinship values were greater than 0.05, indicating that most lines were distantly related. The underlying population structure of these inbred lines was further examined following, model-based clustering. The ΔK analysis indicated a peak at K = 3, suggesting the presence of three distinct genetic subgroups within the panel (**Figure 6**). Genotypes were distributed across multiple subgroups, reflecting the wide genetic base and diverse breeding backgrounds used in their development. In addition, a neighbor-joining (NJ) phylogenetic tree based on genetic distance further confirmed the grouping pattern among the genotypes (**Figure 7**). The population structure (Q matrix) derived from STRUCTURE and the kinship matrix (K matrix), representing genetic relatedness among the lines, were both incorporated into the mixed linear model (MLM) to minimize spurious associations and enhance the accuracy of GWAS.

**Figure 8:**
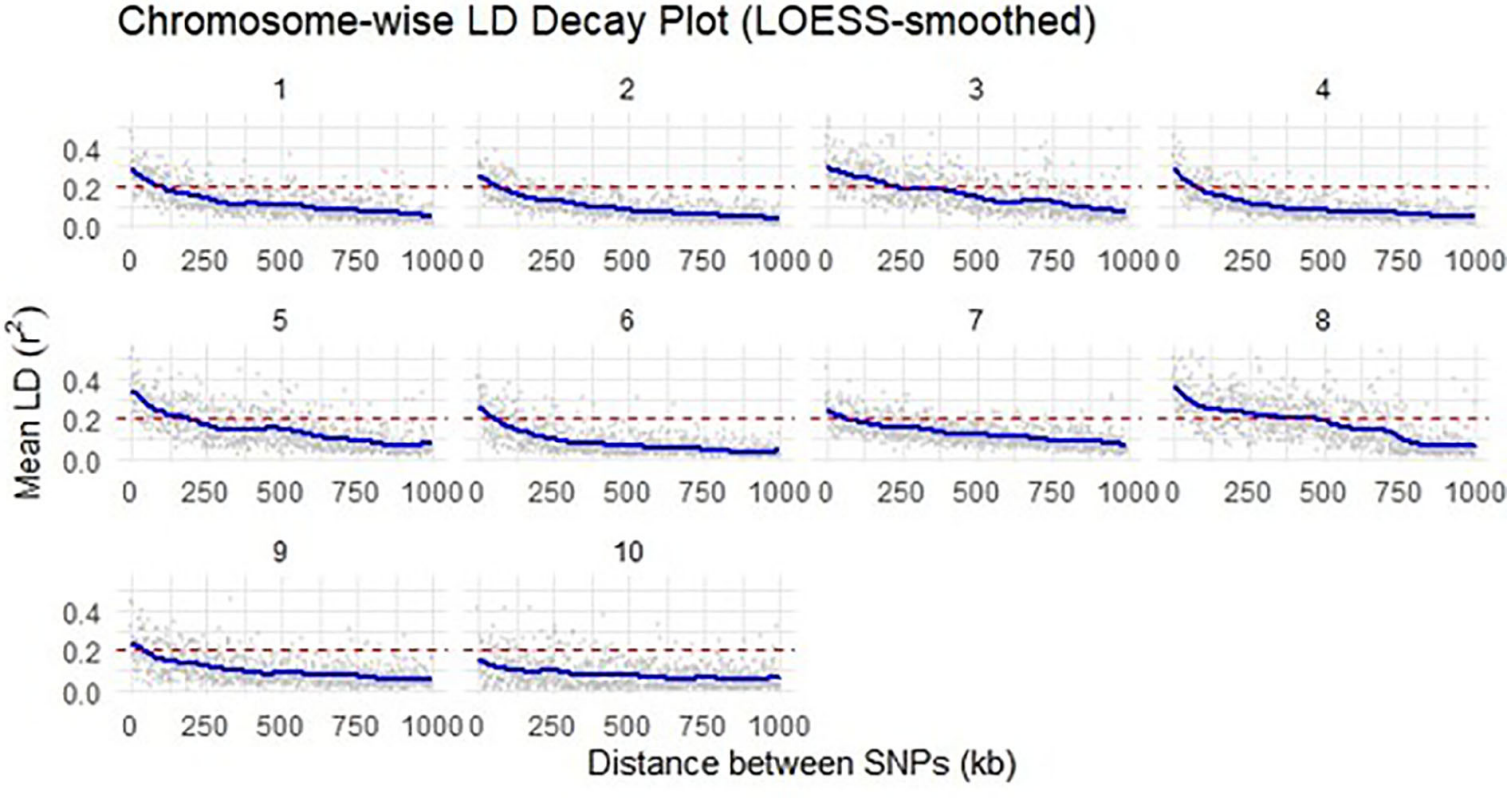
Linkage disequilibrium (LD) decay pattern in maize based on genome-wide SNP markers.

A locally estimated scatter plot smoothing (LOESS) curve facilitated a clearer interpretation of LD decay patterns on individual chromosomes (**Figure 8**). The extent of LD decay varied across chromosomes, with r² values declining more rapidly on certain chromosomes than on others. The estimated distance at which LD decayed ranged from approximately 5 kb on chromosome 10 to 163 kb on chromosome 3 (**Table 3**). The average genome-wide LD decay was estimated at around 65.4 kb, indicating a moderate level of genomic resolution for detecting marker-trait associations in the genome-wide association study.

**Table 3:** Estimated extent of Linkage Disequilibrium (LD) decay (in kilobases) across the chromosomes in the maize association panel.

Chromosome	LD Decay Distance (kb)
1	51
2	110
3	163
4	55
5	68
6	36
7	42
8	105
9	19
10	5

LD, linkage disequilibrium.

### 3.5 Marker trait associations for root architectural traits under aluminum stress

**Figure 9:**
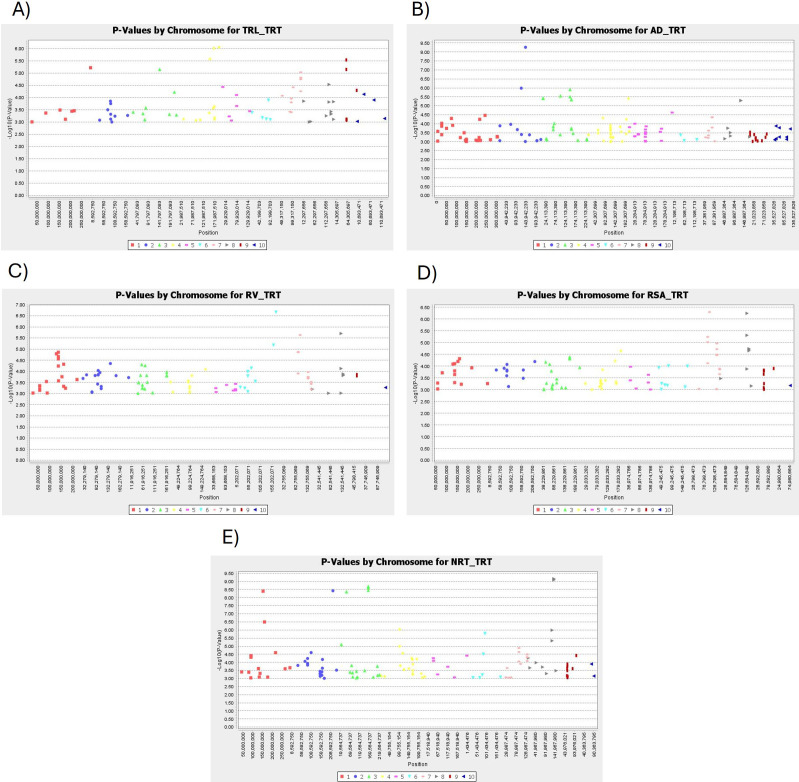
Genome wide association studies for root architectural traits under the Al stress conditions. GWAS results for Total Root Length **(A)**, Average Diameter **(B)**, Root Volume **(C)** and Root Surface Area **(D)**, Number of Root Tips **(E)**.

Following the phenotypic characterization, GWAS analysis for Al tolerance using MLM detected a total of 44 SNPs significantly associated with the five root traits *viz*., TRL (8 SNPs), NRT (15 SNPs), RV (4 SNPs), AD (12 SNPs) and RSA (5 SNPs) at a significance threshold of -log_10_ (p) > 5, distributed across eight chromosomes except on chromosome 5 and 10 (**Table 4**, **Figure 9**).Of the total, 40 MTAs were unique, while three markers were shared across multiple traits, potentially reflecting pleiotropy. These SNPs explained the phenotypic variation from 9.68% to 18.43%. Besides, the largest number of significant SNPs was on the chromosome 3 (13 SNPs) and NRT trait had the most associated SNPs (15 SNPs) (**Table 5**). Further trait-wise detailed MTA results have been presented below.

**Table 4:** Number of MTAs for root traits under aluminum stress in maize at a significance threshold of –log10(p) > 5.

Trait	No. of MTAs	Range of –log10 (p)	Range of PVE (%)	Chromosomes Involved
TRL	8	5.04 – 6.06	9.75 – 11.84	1, 3, 4, 7, 9
NRT	15	5.10 – 9.16	9.85 – 18.43	1, 2, 3, 4, 6, 8
RV	4	5.18 – 6.67	9.98 – 13.01	6, 7, 8
AD	12	5.29 – 8.26	10.34 – 16.61	2, 3, 4, 8
RSA	5	5.02 – 6.30	9.68 – 12.29	7, 8

TRL, total root length (cm); RSA, root surface area (cm²); AD, average root diameter (mm); RV, root volume (cm³), and number of root tips (NRT); PVE (%), phenotypic variance explained; MTAs, marker trait association.

**Table 5:** Detailed list of significant marker–trait associations (MTAs) identified for root architectural traits under aluminum stress in maize.

S.no	Trait	Marker	Chr	Position	p-value	–log_10_ (p)	PVE (%)
1	TRL	SChr4_192058496	4	192058496	8.67E-07	6.06	11.84
2		SChr4_169157503	4	169157503	9.45E-07	6.02	11.76
3		SChr4_151164785	4	151164785	2.61E-06	5.58	10.86
4		SChr9_57274368	9	57274368	2.85E-06	5.55	10.78
5		SChr1_291407250	1	291407250	5.98E-06	5.22	10.12
6		SChr9_57274340	9	57274340	7.00E-06	5.15	9.98
7		SChr3_144190853	3	144190853	7.05E-06	5.15	9.97
8		SChr7_137019441	7	137019441	9.09E-06	5.04	9.75
9	NRT	SChr8_136743030	8	136743030	6.91E-10	9.16	18.43
10		SChr8_136742944	8	136742944	6.97E-10	9.16	18.42
11		SChr8_136742809	8	136742809	8.05E-10	9.09	18.28
12		SChr3_162691279	3	162691279	2.04E-09	8.69	17.40
13		SChr3_162691266	3	162691266	2.37E-09	8.63	17.26
14		SChr3_162691487	3	162691487	3.59E-09	8.44	16.87
15		SChr2_220362889	2	220362889	3.78E-09	8.42	16.82
16		SChr1_153597880	1	153597880	4.04E-09	8.39	16.76
17		SChr3_50815944	3	50815944	4.41E-09	8.36	16.68
18		SChr1_160784456	1	160784456	3.20E-07	6.49	12.73
19		SChr4_103467158	4	103467158	9.16E-07	6.04	11.78
20		SChr8_127618532	8	127618532	1.03E-06	5.99	11.68
21		SChr6_92882553	6	92882553	1.66E-06	5.78	11.25
22		SChr8_127618207	8	127618207	4.64E-06	5.33	10.34
23		SChr3_24883074	3	24883074	7.99E-06	5.10	9.86
24	RV	SChr6_172447002	6	172447002	2.15E-07	6.67	13.01
25		SChr8_127618532	8	127618532	1.99E-06	5.70	11.03
26		SChr7_102943539	7	102943539	2.33E-06	5.63	10.89
27		SChr6_161539730	6	161539730	6.55E-06	5.18	9.98
28	AD	SChr2_142613102	2	142613102	5.46E-09	8.26	16.61
29		SChr2_121055108	2	121055108	1.04E-06	5.98	11.76
30		SChr3_147147013	3	147147013	1.26E-06	5.90	11.59
31		SChr3_98868452	3	98868452	2.84E-06	5.55	10.86
32		SChr3_147146931	3	147146931	3.16E-06	5.50	10.76
33		SChr3_11527008	3	11527008	3.73E-06	5.43	10.62
34		SChr4_210113698	4	210113698	3.89E-06	5.41	10.58
35		SChr3_11526962	3	11526962	3.92E-06	5.41	10.57
36		SChr3_154019446	3	154019446	4.53E-06	5.34	10.44
37		SChr3_154019479	3	154019479	4.53E-06	5.34	10.44
38		SChr8_131335005	8	131335005	5.10E-06	5.29	10.34
39		SChr8_131335018	8	131335018	5.10E-06	5.29	10.34
40	RSA	SChr7_102943539	7	102943539	5.03E-07	6.30	12.29
41		SChr8_127618532	8	127618532	5.70E-07	6.24	12.18
42		SChr8_127618207	8	127618207	4.89E-06	5.31	10.26
43		SChr7_94466659	7	94466659	5.72E-06	5.24	10.13
44		SChr7_94466679	7	94466679	9.55E-06	5.02	9.68

TRL, total root length (cm); RSA, root surface area (cm²); AD, average root diameter (mm); RV, root volume (cm³), and number of root tips (NRT); PVE (%), phenotypic variance explained; MTAs, marker trait associations; chr, chromosome.

#### 3.5.1 TRL (total root length)

TRL trait has been associated with eight SNPs distributed across five chromosomes *viz*., 1, 3, 4, 7 and 9 (**Table 5**, **Figure 9**). Chromosome 4 has the highest number of the SNPs harboring three SNPs followed by chromosome 9 with two SNPs and chromosomes 3, 7 and 1 with one SNP each. These markers showed the PVE ranging from 9.75% to 11.84%. SNP marker SChr4_192058496 had the most significant association (–log10 (p) = 6.06) and accounted for the largest proportion of variation (PVE = 11.84%), indicating its potential relevance for maintaining trait under aluminum toxicity. Further two SNPs viz., SChr9_57274368 and SChr9_57274340 were closely located on the chromosome 9 within a distance of 28 base pairs suggesting a possible genomic hotspot for the TRL trait.

#### 3.5.2 NRT (number of root tips)

GWAS identified 15 SNPs associated with the NRT trait distributed across six chromosomes *viz*., 1, 2, 3, 4, 6 and 8 (**Table 5**, **Figure 9**). Chromosomes 8 and 3 had the highest number of SNPs with 5 SNPs each followed by chromosomes 1 with 2 SNPs and chromosomes 2, 4 and 6 with 1 SNP each. These markers showed PVE% ranging from 9.86% to 18.43%, with SChr8_136743030 explaining the maximum variation (18.43%). Three SNPs on chromosome 8 (SChr8_136743030, SChr8_136742944, and SChr8_136742809) were located within a narrow window of 221 bp, while another cluster of three SNPs on chromosome 3 (SChr3_162691279, SChr3_162691266, and SChr3_162691487) also mapped within 221 bp. These clusters highlight potential genomic hotspots associated with NRT.

#### 3.5.3 RV (root volume)

A total of 4 SNPs were associated with the RV trait distributed across three chromosomes viz., 1, 6, 7 and 8 (**Table 5**, **Figure 9**). Chromosome 6 has the highest number of SNPs with 2 SNPs followed by chromosome 7 and 8 with 1 SNP each. These markers showed PVE ranging from 9.98% to 13.01%, with SNP marker SChr6_172447002 explaining the maximum variation (PVE = 13.01%).

#### 3.5.4 AD (average root diameter)

A total of twelve SNPs were associated with the AD trait distributed across four chromosomes viz., 2, 3, 4, and 8 (**Table 5**, **Figure 9**). Chromosome 3 has the highest number of SNPs with 7 SNPs followed by chromosome 7and 8 with 2 SNP each and chromosome 4 with 1 SNP. These markers showed PVE ranging from 10.34% to 16.61%, with SNP marker SChr2_142613102 explaining the maximum variation (PVE = 16.61%).Several closely linked SNP pairs identified are indicating the possible genomic hotspots. SNPs SChr3_147147013 and SChr3_147146931 (82 bp apart), SChr3_11527008 and SChr3_11526962 (46 bp apart), SChr3_154019446 and SChr3_154019479 (33 bp apart), and SChr8_131335005 and SChr8_131335018 (13 bp apart). Collectively, these clusters represent multiple potential hotspots on chromosomes 3 and 8 for AD.

#### 3.5.5 RSA (root surface area)

For RSA, five SNPs were associated with the RSA trait distributed across two chromosomes *viz*., 7 and 8 (**Table 5**, **Figure 9**). Chromosome 7 had the highest number of the SNPs with 3 SNPs followed by chromosome 8 with 2 SNPs. These markers showed the PVE ranging from 9.68% to 12.29%, with SNP marker SChr7_102943539 accounting for the largest proportion of variation (12.29%). Two pairs of SNPs, SChr7_94466659 and SChr7_94466679 (20 bp apart) and SChr8_127618532 and SChr8_127618207 (325 bp apart), were closely positioned, suggesting putative genomic hotspots for RSA on both chromosomes.

### 3.6 QTL hotspots for the root architectural traits under aluminum stress

**Figure 10:**
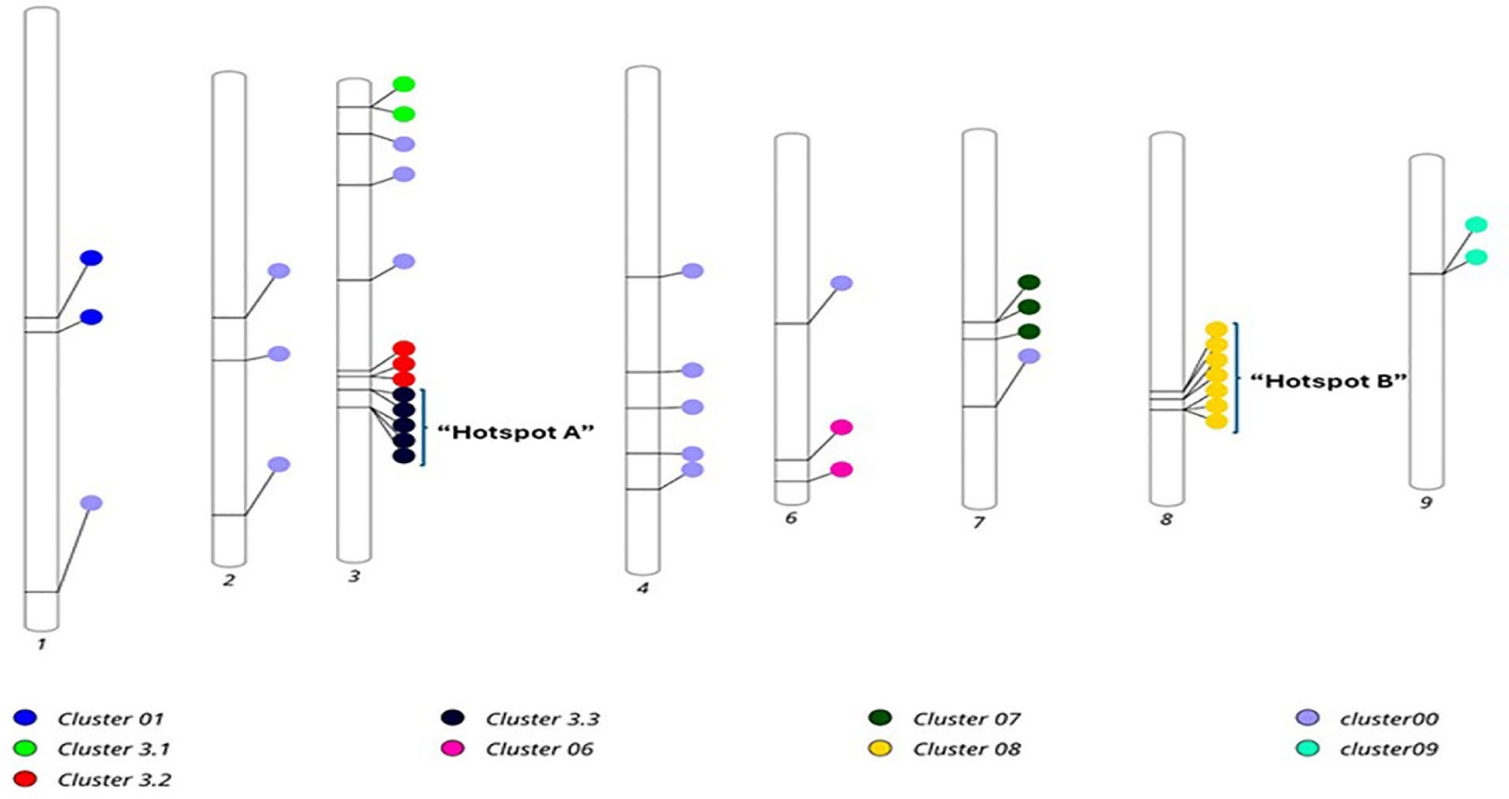
Distribution of 40 MTAs, eight QTL clusters (Cluster -01, Cluster-3.1, Cluster -3.2, Cluster -3.3, Cluster -06, Cluster -07, Cluster -08, and Cluster -09) and two hotspots (Hotspot A and Hotspot B) on different chromosomes identified for root architectural traits under Al stress conditions.

GWAS identified several chromosomal regions where significant SNPs clustered, representing potential genomic hotspots for root trait regulation. In this study, we found eight QTL clusters on six different chromosomes *viz*., Chr1, Chr3, Chr6, Chr7, Chr8 and Chr9 and were named as Cluster-01, Cluster-3.1, Cluster -3.2, Cluster -3.3, Cluster -06, Cluster -07, Cluster -08 and Cluster -09 respectively (**Table 6**, **Figure 10**). The highest concentration of the QTLs for root traits under Al stress was identified in Cluster-08 of Chr8 and is designated as “QTL hot spot A” spanning a physical length of 9.1 Mb. This QTL hot spot harbors seven QTLs *viz*., SChr8_127618207, SChr8_127618532, SChr8_131335005, SChr8_131335018, SChr8_136742809, SChr8_136742944 and SChr8_136743030 associated to NRT (5 SNPs), RSA (2 SNPs), RV (1 SNPs) and AD (2 SNPs) (**Table 6**). All the QTLs underlying “QTL hot spot A” were major QTLs (PVE > 10%) and explained 10.26% to 18.43% of the phenotypic variation (**Table 6**). Another set of QTL rich region was found in Cluster 3.3 on Chr 3 and is designated as “QTL hot spot B” with length of 8.7 Mb. It harbors five QTLs for NRT (3 SNPs) and AD (2 SNPs) traits that include SChr3_154019446, SChr3_154019479, SChr3_162691266, SChr3_162691279 and SChr3_162691487. All the QTLs underlying “QTL hot spot B” were major QTLs (PVE > 10%) and explained 10.44% to 17.40% of the phenotypic variation. Clusters 07 and Cluster 3.2 contain three QTLs each explaining phenotypic variation of 9.68% to 12.29% and 9.97% to 11.59% respectively, whereas the remaining clusters viz., Cluster -09, Cluster-3.1, Cluster -01, and Cluster -06 contain 2 SNPs each explaining phenotypic variation of 9.98% to 10.78%, 10.57% to 10.62%, 12.73% to 16.76%, and 9.98% to 13.01% respectively. These are the genomic regions governing the root traits for Al toxicity tolerance in maize.

**Table 6:** Eight QTL clusters identified for root architectural traits under aluminum stress and their chromosomal locations.

QTL cluster name	Physical range	QTLs	PVE %
Cluster -01	153597880 - 160784456	SChr1_153597880	16.76
		SChr1_160784456	12.73
Cluster-3.1	11526962 - 11527008	SChr3_11526962	10.57
		SChr3_11527008	10.62
Cluster -3.2	144190853 - 147147013	SChr3_144190853	9.97
		SChr3_147146931	10.76
		SChr3_147147013	11.59
Cluster -3.3	154019446 - 162691487	SChr3_154019446	10.44
		SChr3_154019479	10.44
		SChr3_162691266	17.26
		SChr3_162691279	17.40
		SChr3_162691487	16.87
Cluster -06	161539730 - 172447002	SChr6_161539730	9.98
		SChr6_172447002	13.01
Cluster -07	94466659 - 102943539	SChr7_94466659	10.13
		SChr7_94466679	9.68
		SChr7_102943539	12.29
Cluster -08	127618207 - 136743030	SChr8_127618207	10.34
		SChr8_127618532	11.68
		SChr8_131335005	10.34
		SChr8_131335018	10.34
		SChr8_136742809	18.28
		SChr8_136742944	18.42
		SChr8_136743030	18.43
Cluster -09	57274340 - 57274368	SChr9_57274340	9.98
		SChr9_57274368	10.78

### 3.7 Candidate genes underlying the genomic regions for root architectural traits under aluminum stress

**Figure 11:**
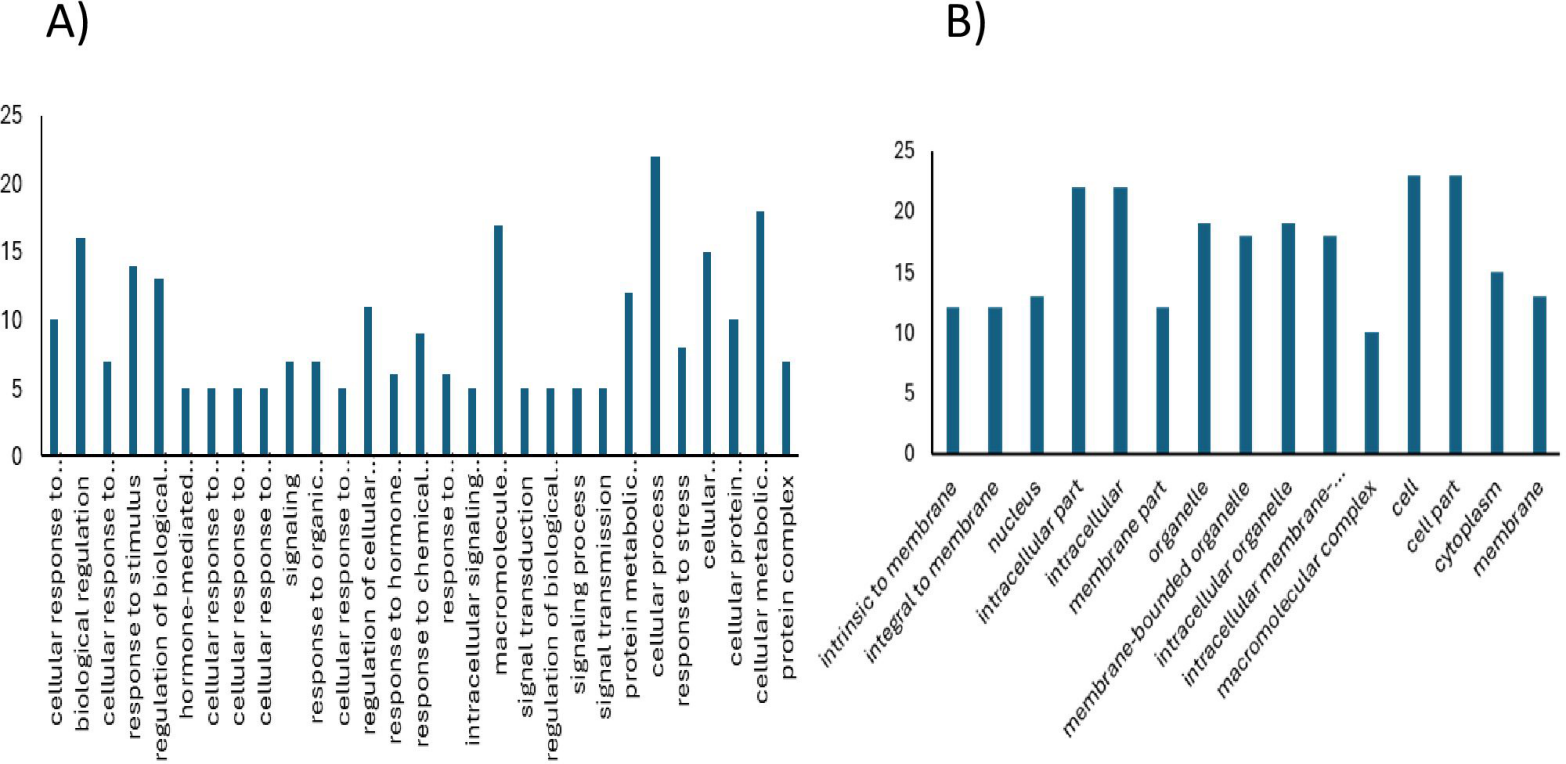
Functional categories of candidate genes in 65.4 kb flanking regions around peak SNPs. **(A)** Biological process **(B)** cellular component.

The candidate genes were searched against the maize reference genome (B73). The average LD decay rate estimated in this population is approximately 65.4 kb, we applied 65.4 kb window on either side of the significant SNP to investigate the local LD pattern and search for putative candidate genes. A total of 61 genes were found in these regions after excluding the uncharacterized proteins, among which, 28 genes had unknown functions. The remaining 33 genes were functionally characterized and considered as potential candidates associated with Al tolerance in maize (**Supplementary Table S2-S6**, **Figure 11**). These were related to antioxidant defense-related enzymes (e.g., glutathione S-transferases, thioredoxins, dehydroascorbate reductases), signal transduction and stress-responsive kinases (e.g., serine/threonine kinases, MAPK pathway components), transcriptional regulators (e.g., bHLH transcription factors, homeobox-leucine zipper proteins, NF-Y subunits), transporters (e.g., auxin efflux carriers, sodium/hydrogen exchangers), RNA-binding and post-transcriptional regulators (e.g., RRM domain-containing proteins, mRNA cap-binding proteins), and proteolytic machinery components (e.g., F-box proteins, aspartyl proteases) and other functions. Among the identified candidate genes, *Zm00001eb355550*and *Zm00001eb354260*, both encoding glutathione S-transferases (GSTs), co-localized on chromosome 8, in proximity to SChr8_136743030 and SChr8_136742944, as well as SChr8_131335005 and SChr8_131335018. GSTs help in detoxification and scavenging the reactive oxygen species (ROS), specifically in root tissues, thereby helps the defense system under the oxidative stress induced by abiotic factors such as Al toxicity.*Zm00001eb355540*, located near SChr8_136743030and SChr8_136742944 on chromosome 8, encodes Dehydroascorbate reductase-like1 (DHAR-like1) protein. DHAR enzymes are vital components of ascorbate - glutathione cycle maintains the redox balance in the root cells by regulating the reduced ascorbate from its oxidized form to strengthen the antioxidant defense system, thereby protecting the root cells. *Zm00001eb125730*, located near SChr3_24883074 on chromosome 3 encodes a heavy metal transport/detoxification protein, which includes members of the *MATE* (Multidrug and Toxic Compound Extrusion), *ABC* (ATP-binding cassette), and *P-type ATPase* transporter families. These transporters play a central role in aluminum (Al) and heavy metal detoxification by mediating the efflux or vacuolar sequestration of toxic ions, thereby maintaining cellular ion homeostasis and enhancing stress resilience. Additionally, *Zm00001eb293930* and *Zm00001eb310980*, located near SChr6_172447002 and SChr7_102943539 on chromosomes 6 and 7, encode an F-box domain-containing protein and THO Complex Subunit 6 (THO6), respectively. Both proteins are functionally linked to the regulation of the transcription factor STOP1, a central regulator of Al-inducible genes such as ALMT. Through ubiquitin-mediated degradation, the F-box protein modulates STOP1 stability, while THO6 contributes to its transcriptional and post-transcriptional regulation, together highlighting their importance in fine-tuning STOP1 activity and thereby enhancing aluminum stress tolerance. Among the identified candidate genes, these six genes showed stronger evidence based on their functional annotations and biological relevance and therefore may be considered as the most promising candidates for conferring Al stress tolerance in maize.

### 3.8 *In-silico* expression analysis of promising candidate genes under the abiotic stress

**Figure 12:**
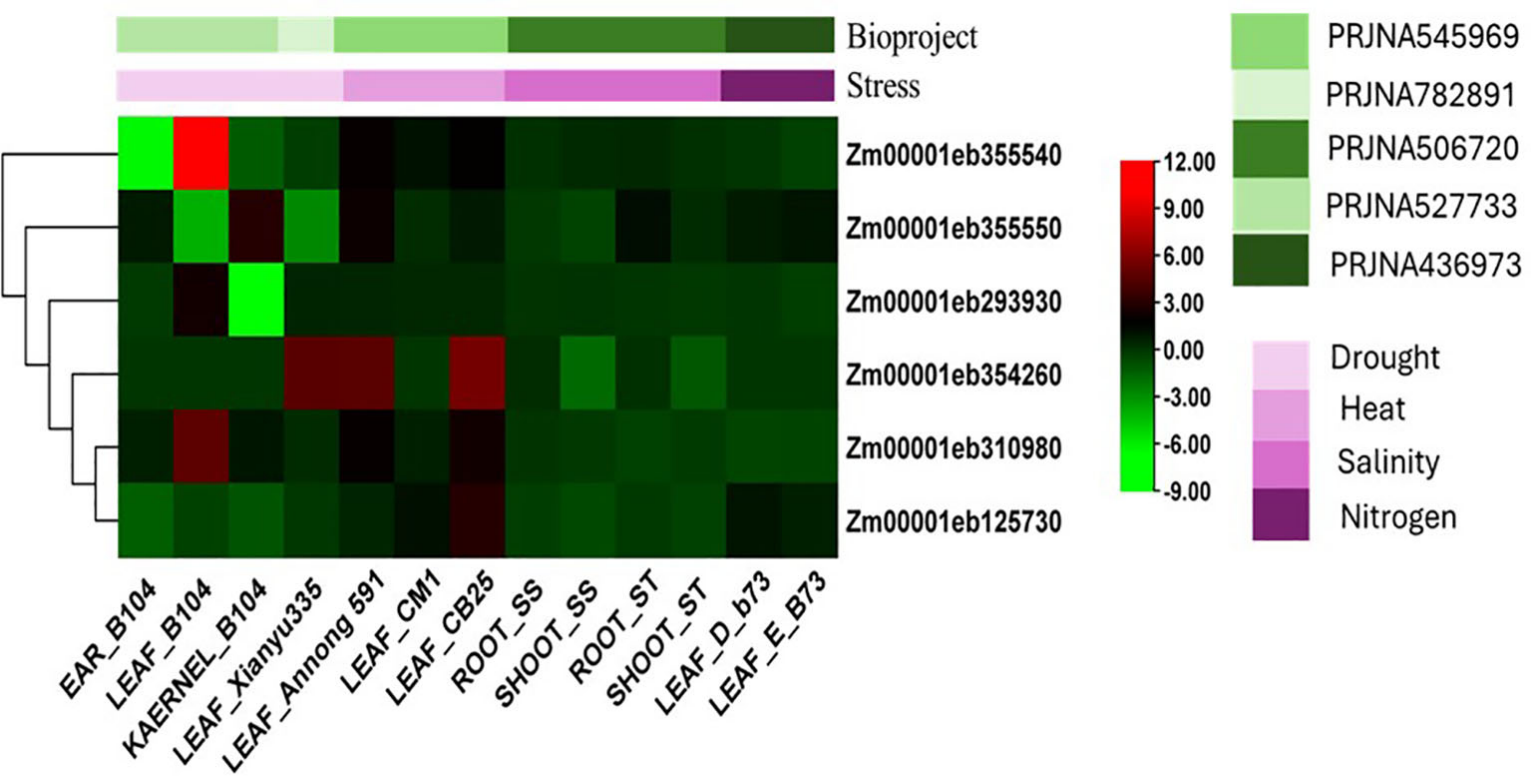
*In-silico* expression of selected candidate genes in response to various abiotic stresses in maize: Heat maps showing expression of candidate genes in maize during drought, heat, salinity, and nitrogen stresses. The data shows log2fold values of expression. The fold change values of each gene expression were computed through comparing with plants grown under control or optimum environment.

We further explored the functional relevance of the promising genes through *in-silico* expression profiling using public transcriptome datasets across diverse maize genotypes and tissues (**Figure 12**). Under drought stress, the gene *Zm00001eb355540* was strongly induced in B104 leaves (10.02) but suppressed in ears (–6.36) and kernels (–1.18). *Zm00001eb310980* (4.75) and *Zm00001eb293930* (2.35) were also up regulated in B104 leaves, while *Zm00001eb355550* showed induction in kernels (2.94) but repression in leaves (–3.98). *Zm00001eb125730* was consistently down regulated across tissues (–0.39 to –1.30). Under heat stress, *Zm00001eb354260* showed strong induction in leaves of Xianyu335 (4.61), Annong591 (4.64), and CB25 (5.57). *Zm00001eb310980* (B104: 4.75; CB25: 2.28) and *Zm00001eb355540* (Annong591: 1.96; CB25: 1.87) were also highly expressed, while *Zm00001eb125730* was moderately induced (up to 2.97). *Zm00001eb355550* showed lower expression (0.29–2.14).

Under salinity stress, expression was generally low. *Zm00001eb355550* was induced in ST roots (1.29) but repressed in SS roots (–0.10). *Zm00001eb354260* (0.17–0.29) and *Zm00001eb293930* (0.02–0.15) showed weak induction, while *Zm00001eb125730* was suppressed in both roots (–0.12 to –0.23) and shoots (–0.35 to –0.61). Under the nitrogen starvation, *Zm00001eb355550* (0.84–1.07) and *Zm00001eb125730* (0.76–1.09) were up regulated in B73 leaves. *Zm00001eb310980* (–0.44 to –0.47) and *Zm00001eb293930* (–0.28 to 0.03) were repressed, whereas *Zm00001eb354260* showed no detectable expression.

## 4 Discussion

### 4.1 Protocol standardization and phenotypic characterization of root traits under aluminum stress

The present study established a robust hydroponic protocol for Aluminium stress screening and provided insights into the phenotypic responses of root traits in maize. Aluminium toxicity is known to target the root apex and meristematic tissues, rapidly disrupting cell division and elongation, which in turn suppresses both primary root extension and lateral root initiation. This was reflected in our results, where TRL and NRT emerged as the most sensitive indicators of toxicity. The strong reduction in these traits highlights their central role in nutrient and water uptake and explains the sharp decline in root elongation and branching under stress. Similar observations have been reported previously, emphasizing that moderate levels of Al stress most effectively reveal genotypic differences, whereas severe stress obscures variation by uniformly suppressing root growth (Magnavaca et al., 1987; Chaffai et al., 2005). In agreement, our screening revealed that very high concentrations (≥600 μM) were detrimental across all genotypes, while a moderate level (300 μM AlCl_3_) captured clear contrasts, particularly when evaluated on day 11. This time point coincided with the stabilization of stress responses, reflecting the lag required for physiological mechanisms such as organic acid exudation, callose deposition, and cell wall modification to take effect (Famoso et al., 2010; Coelho et al., 2015). Thus, the combination of 300 μM AlCl_3_ and evaluation at day 11 provided an optimal balance between stress intensity and resolution, aligning with established hydroponic standards (Magnavaca et al., 1987; Famoso et al., 2010). Beyond protocol optimization, trait-specific responses shed light on the mechanisms of Al tolerance. TRL consistently served as a primary diagnostic trait, while RSA and NRT, which represent surface expansion and fine root proliferation, were also strongly inhibited, confirming earlier evidence that Al interferes with lateral root branching (Kochian et al., 2004). In contrast, AD increased under stress, suggesting a compensatory adjustment rather than a direct tolerance mechanism. Such thickening responses, often associated with radial cell expansion and cortical reinforcement, were more pronounced in tolerant genotypes, implying that they complement rather than replace elongation and branching responses (Kollmeier et al., 2000; Chaffai et al., 2005). RV acted as a composite measure of root growth, but its broad variability and occasional hypertrophy limited its utility as a standalone indicator (BarcelóPoschenrieder, 2002). These distinctions collectively underscore that Al tolerance is a multifaceted trait requiring coordinated preservation of elongation and branching supported by secondary morphological adjustments.

Correlation analysis further highlighted this coordination. Strong positive associations between TRL and NRT (r = 0.92), and moderate correlations with RSA (r = 0.72) and RV (r = 0.54), suggest that elongation, branching, and surface traits form a tightly linked response network under Al stress. This agrees with reports that root tip integrity and branching capacity are interdependent determinants of tolerance (Kochian et al., 2004). In contrast, the weak correlation between TRL and AD (r = 0.06) reinforced the independence of thickening as a secondary adjustment mechanism (Zhao et al., 2019). Strong correlations of TRL with RSA, NRT, and RV indicate that these elongation and branching traits act as a unified root architecture module and can be prioritized for selection under Al stress, whereas AD, being weakly correlated, represents a secondary compensatory response rather than a primary selection trait. Together, these findings demonstrate that effective screening requires both optimized stress protocols and a trait-based framework that integrates elongation, branching, and compensatory thickening responses, thereby providing a comprehensive foundation for dissecting genetic mechanisms of Al tolerance in maize.

### 4.2 Marker trait associations and hotspots for root architectural traits under aluminum stress

To our knowledge, this was the first study to genetically dissect the Al stress tolerance based on root traits by GWAS using the high-density SNPs in maize. Compared with QTL mapping using the biparental populations, GWAS harnesses broader genetic diversity, and potentially has higher mapping resolutions, which can facilitate the identification of candidate genes in the single experiment (Yano et al., 2016). Although QTLs related to Al tolerance have been identified in various crops such as wheat (Navakode et al., 2014), rice (Famoso et al., 2011; Tao et al., 2018), common bean (Ambachew and Blair, 2021), and rapeseed (Gao et al., 2020; Zhou et al., 2022), the genetic information available in maize remains limited. Earlier studies using traditional bi-parental mapping approaches have reported a total of 14QTLs associated with Al stress tolerance distributed across chromosomes *viz*., 2, 4, 5, 6, 7, 8, 9 and 10 (Sibov et al., 1999; Ninamango-Cárdenas et al., 2003; Conceição et al., 2009; Coelho et al., 2019) in maize.

In our study, we identified 40 unique and significant SNPs (–log_10_p > 5) associated with five root traits under Al stress, distributed across eight out of ten maize chromosomes except chr. 5 and chr 10. Comparison with earlier studies revealed that our root-associated QTLs under Al toxicity overlap with previously reported QTLs on chromosomes 2, 4, 6, 7, 8 and 9 (Sibov et al., 1999; Ninamango-Cárdenas et al., 2003; Conceição et al., 2009; Coelho et al., 2019). Additionally, we identified eight QTL clusters including two hotspots on six different chromosomes *viz*., Chr1, Chr3, Chr6, Chr7, Chr8, and Chr9 associated with Al tolerance. “QTL hot spot A” on Chr8 spanning physical length of 9.1 Mb contains seven QTLs. Ninamango-Cárdenas et al. (2003) and Conceição et al. (2009) had previously reported the QTLs on the chr8 providing further support for the involvement of these genomic regions in Al stress response. Despite some chromosomal overlaps with previously reported QTLs, “QTL hot spot B” on chr3 spanning physical length of 8.7 Mb contains five QTLs emerges out as the novel QTLs rich region for Al stress tolerance with no previous studies reported the QTLs on chr3. These SNP clusters likely represent localized LD blocks within the identified QTL regions, and a haplotype-based approach may help refine these loci further in future studies. The novelty of our findings can be attributed to the high-resolution power of SNP-based GWAS, which offers improved detection of allelic variants compared to earlier SSR-based studies (Korte and Farlow, 2013). Thus, our results suggest that the genomic regions on chr8 and chr3 represent the hotspots of genetic for Al stress tolerance in maize. Future studies should prioritize these regions for fine mapping, candidate gene validation, and incorporation into breeding programs through marker-assisted selection (MAS) and genomic prediction.

### 4.3 Candidate genes underlying the genomic regions for root architectural traits under aluminum stress

To date, no studies have identified candidate genes regulating Al stress in tropical maize using genome-wide association studies (GWAS). Aluminum toxicity is a complex trait influenced by multiple physiological, biochemical and molecular processes, including oxidative stress responses, hormonal signaling, membrane stability, and protein homeostasis. In this study, we employed GWAS to identify significant SNPs and the corresponding candidate genes associated with Al stress tolerance. A total of 33 candidate genes were identified within the genomic regions surrounding significant SNPs, many of which are associated with stress-responsive pathways. Because Al tolerance in plants is known to involve several well-characterized mechanisms such as (i) organic acid exudation that chelates apoplastic Al³^+^, mediated by transporters like MATE, ALMT, and ABC families (ii) modification of the cell wall to reduce Al binding (iii) transporter-mediated exclusion or sequestration of Al ions (iv) antioxidant and ROS-detoxification pathways that mitigate Al-induced oxidative stress; and (v) transcriptional and post-transcriptional regulation controlled by factors such as STOP1. The functional annotation of the identified genes was interpreted within the framework of these established Al-tolerance pathways. Among the identified candidates, we focused on genes with mechanistic roles in aluminum toxicity tolerance.

Aluminum toxicity is closely associated with oxidative stress, as Al³^+^ induces concentration and time dependent ROS accumulation and protein oxidation in root tissues (Boscolo et al., 2003). In maize, Cañçado et al. (2005) showed that increased expression of the Al-responsive *Gst27.2* gene helps limit ROS build-up, thereby reducing the need for other antioxidant enzymes. In our study, the candidate genes *Zm00001eb355550 andZm00001eb354260*, both encoding glutathione S-transferases (GSTs), were located within major Al-associated QTL regions on chromosome 8. Given the established role of GSTs in detoxifying ROS and maintaining redox balance, these genes likely contribute to mitigating Al-induced oxidative damage and supporting root growth under Al stress. Another candidate, *Zm00001eb355540*, annotated as *Dehydroascorbate reductase-like1* (DHAR-like1), was identified near SNPs associated with the number of root tips (NRT). DHARs are key components of the ascorbate–glutathione cycle, regenerating reduced ascorbate and maintaining cellular redox balance. Over expression of DHAR in transgenic tobacco enhanced root growth under Al stress by reducing ROS accumulation and oxidative damage rather than decreasing Al uptake (Yin et al., 2010).The candidate gene *Zm00001eb125730*, associated with NRT on chromosome 3, encodes a heavy metal transport/detoxification protein that likely belongs to transporter families such as MATE, ABC, or P-type ATPases. Members of these transporter families play central roles in aluminum tolerance by facilitating ion efflux across the plasma membrane or by sequestering toxic Al complexes into intracellular compartments, thereby reducing cytosolic toxicity (Paape et al., 2022). Well-characterized examples of this mechanism include *SbMATE* in sorghum and *HvAACT* in barley, which mediate Al-activated citrate efflux to chelate Al³^+^ in the rhizosphere (Magalhães et al., 2007; Furukawa et al., 2007). In maize, *ZmMATE1* functions similarly, and in rice, *OsFRDL4* controls citrate secretion from root cells to enhance external detoxification (Maron et al., 2010; Yokosho et al., 2014). Comparable MATE-mediated organic acid efflux mechanisms have also been reported in soybean and buckwheat (Zhou et al., 2019). Given this functional context, *Zm00001eb125730* may contribute to Al tolerance in the present panel by promoting organic acid efflux or intracellular sequestration of Al ions, thereby aligning with well-established transporter-mediated detoxification pathways.Two additional candidate genes, *Zm00001eb293930* and *Zm00001eb310980*, were detected near SNPs SChr6_172447002 and SChr7_102943539, respectively, encoding an F-box domain protein and *THO Complex Subunit 6 (THO6)*. F-box proteins regulate transcription factor stability via ubiquitin-mediated degradation. In Arabidopsis, the F-box protein *RAE1* modulates Al tolerance by targeting *STOP1*, a key transcription factor for Al-inducible genes such as *AtALMT1*, within a negative feedback loop that adjusts STOP1 stability (Zhang et al., 2019). On the other hand, *THO6* is part of the conserved THO/TREX complex, which plays a critical role in transcription, RNA processing, and mRNA export. This complex contributes to Al tolerance by facilitating pre-mRNA processing, small RNA biogenesis, and transcript export essential for stress-responsive gene expression. Disruption of other THO/TREX components, such as *HPR1*, has been shown to reduce STOP1 accumulation and impair Al detoxification capacity (Guo et al., 2020), underlining the importance of this pathway in Al stress adaptation. Collectively, these candidate genes highlight diverse but interconnected mechanisms underlying maize Al tolerance, ranging from antioxidant defence and redox regulation to transporter-mediated detoxification and transcriptional/post-transcriptional control. Comprehensive functional characterization through gene expression profiling, transgenic approaches, and knockout studies would help elucidate their specific mechanisms of action. Such investigations will strengthen the understanding of the molecular basis of aluminum tolerance and facilitate the development of Al-resilient maize varieties through molecular breeding.

## Data Availability

The original contributions presented in the study are included in the article/**Supplementary Material**. Further inquiries can be directed to the corresponding authors.
