# Supplementary material for: Genetic dissection of seedling root architecture under aluminium toxicity in tropical maize (Zea mays L.)
Source: Front Plant Sci. 2026 Feb 10;16:1722162. doi: 10.3389/fpls.2025.1722162 (PMC12929552; doi:10.3389/fpls.2025.1722162)
Supplement: Supplementary file 1 [file Table1.docx]

**Table S1** List of genotypes in AMP

| Sl.No | Entry | Sl.No | Entry | Sl.No | Entry | Sl.No | Entry | Sl.No | Entry |
| --- | --- | --- | --- | --- | --- | --- | --- | --- | --- |
| 1 | IMR4 | 51 | IMR178 | 101 | IMR363 | 151 | IMR451 | 201 | IMR580 |
| 2 | IMR5 | 52 | IMR179 | 102 | IMR367 | 152 | IMR452 | 202 | IMR581 |
| 3 | IMR8 | 53 | IMR181 | 103 | IMR368 | 153 | IMR454 | 203 | IMR585 |
| 4 | IMR9 | 54 | IMR182 | 104 | IMR373 | 154 | IMR455 | 204 | IMR592 |
| 5 | IMR15 | 55 | IMR185 | 105 | IMR375 | 155 | IMR457 | 205 | IMR594 |
| 6 | IMR17 | 56 | IMR198 | 106 | IMR377 | 156 | IMR458 | 206 | IMR595 |
| 7 | IMR20 | 57 | IMR202 | 107 | IMR378 | 157 | IMR459 | 207 | IMR596 |
| 8 | IMR26 | 58 | IMR203 | 108 | IMR379 | 158 | IMR460 | 208 | IMR599 |
| 9 | IMR27 | 59 | IMR205 | 109 | IMR380 | 159 | IMR462 | 209 | IMR600 |
| 10 | IMR33 | 60 | IMR208 | 110 | IMR383 | 160 | IMR463 | 210 | IMR601 |
| 11 | IMR34 | 61 | IMR209 | 111 | IMR384 | 161 | IMR464 | 211 | IMR602 |
| 12 | IMR42 | 62 | IMR212 | 112 | IMR385 | 162 | IMR470 | 212 | IMR603 |
| 13 | IMR43 | 63 | IMR220 | 113 | IMR386 | 163 | IMR473 | 213 | IMR604 |
| 14 | IMR44 | 64 | IMR228 | 114 | IMR388 | 164 | IMR476 | 214 | IMR606 |
| 15 | IMR49 | 65 | IMR230 | 115 | IMR390 | 165 | IMR477 | 215 | IMR608 |
| 16 | IMR50 | 66 | IMR233 | 116 | IMR391 | 166 | IMR479 | 216 | IMR609 |
| 17 | IMR55 | 67 | IMR234 | 117 | IMR392 | 167 | IMR482 | 217 | IMR611 |
| 18 | IMR58 | 68 | IMR236 | 118 | IMR393 | 168 | IMR483 | 218 | IMR613 |
| 19 | IMR60 | 69 | IMR241 | 119 | IMR394 | 169 | IMR485 | 219 | IMR614 |
| 20 | IMR63 | 70 | IMR243 | 120 | IMR395 | 170 | IMR486 | 220 | IMR615 |
| 21 | IMR76 | 71 | IMR244 | 121 | IMR396 | 171 | IMR487 | 221 | IMR616 |
| 22 | IMR82 | 72 | IMR245 | 122 | IMR397 | 172 | IMR488 | 222 | IMR617 |
| 23 | IMR87 | 73 | IMR246 | 123 | IMR399 | 173 | IMR490 | 223 | IMR619 |
| 24 | IMR97 | 74 | IMR247 | 124 | IMR401 | 174 | IMR493 | 224 | IMR620 |
| 25 | IMR98 | 75 | IMR249 | 125 | IMR402 | 175 | IMR494 | 225 | IMR621 |
| 26 | IMR99 | 76 | IMR256 | 126 | IMR405 | 176 | IMR495 | 226 | IMR622 |
| 27 | IMR100 | 77 | IMR272 | 127 | IMR406 | 177 | IMR497 | 227 | IMR623 |
| 28 | IMR102 | 78 | IMR277 | 128 | IMR408 | 178 | IMR500 | 228 | IMR624 |
| 29 | IMR104 | 79 | IMR278 | 129 | IMR416 | 179 | IMR505 | 229 | IMR626 |
| 30 | IMR105 | 80 | IMR279 | 130 | IMR420 | 180 | IMR508 | 230 | IMR627 |
| 31 | IMR109 | 81 | IMR291 | 131 | IMR421 | 181 | IMR509 | 231 | IMR629 |
| 32 | IMR114 | 82 | IMR292 | 132 | IMR422 | 182 | IMR518 | 232 | IMR630 |
| 33 | IMR116 | 83 | IMR293 | 133 | IMR424 | 183 | IMR519 | 233 | IMR631 |
| 34 | IMR127 | 84 | IMR294 | 134 | IMR425 | 184 | IMR520 | 234 | IMR632 |
| 35 | IMR128 | 85 | IMR295 | 135 | IMR427 | 185 | IMR532 | 235 | IMR633 |
| 36 | IMR130 | 86 | IMR296 | 136 | IMR428 | 186 | IMR534 | 236 | IMR636 |
| 37 | IMR136 | 87 | IMR297 | 137 | IMR429 | 187 | IMR537 | 237 | IMR637 |
| 38 | IMR138 | 88 | IMR298 | 138 | IMR430 | 188 | IMR538 | 238 | IMR638 |
| 39 | IMR139 | 89 | IMR300 | 139 | IMR431 | 189 | IMR540 | 239 | IMR640 |
| 40 | IMR145 | 90 | IMR303 | 140 | IMR436 | 190 | IMR543 | 240 | IMR641 |
| 41 | IMR149 | 91 | IMR313 | 141 | IMR437 | 191 | IMR546 | 241 | IMR653 |
| 42 | IMR150 | 92 | IMR322 | 142 | IMR439 | 192 | IMR560 | 242 | IMR654 |
| 43 | IMR152 | 93 | IMR325 | 143 | IMR440 | 193 | IMR561 | 243 | IMR655 |
| 44 | IMR156 | 94 | IMR327 | 144 | IMR441 | 194 | IMR562 | 244 | IMR663 |
| 45 | IMR157 | 95 | IMR332 | 145 | IMR442 | 195 | IMR569 | 245 | IMR664 |
| 46 | IMR158 | 96 | IMR342 | 146 | IMR444 | 196 | IMR571 | 246 | IMR665 |
| 47 | IMR169 | 97 | IMR349 | 147 | IMR445 | 197 | IMR573 | 247 | IMR666 |
| 48 | IMR171 | 98 | IMR357 | 148 | IMR446 | 198 | IMR574 | 248 | IMR669 |
| 49 | IMR172 | 99 | IMR358 | 149 | IMR447 | 199 | IMR576 | 249 | IMR670 |
| 50 | IMR175 | 100 | IMR360 | 150 | IMR450 | 200 | IMR579 | 250 | IMR672 |
