# Supplementary material for: Genetic dissection of seedling root architecture under aluminium toxicity in tropical maize (Zea mays L.)
Source: Front Plant Sci. 2026 Feb 10;16:1722162. doi: 10.3389/fpls.2025.1722162 (PMC12929552; doi:10.3389/fpls.2025.1722162)
Supplement: Supplementary file 2 [file Table2.docx]

**Table S2 Putative candidate genes and molecular functions in the 65.4 kb region of linked SNPs for Total Root Length (TRL) under aluminium stress**

| **Trait** | **SNP** | **Chro** | **Position** | **Transcript ID** | **Protein** | **Role** | **Reference(s)** |
| --- | --- | --- | --- | --- | --- | --- | --- |
| **TRL** | SChr4_192058496 | 4 | 192058496 | Zm00001eb196230 | RRM domain-containing protein (RNA-binding motif) | RNA-binding protein involved in mRNA/rRNA processing, RNA transport, stability, and ROS detoxification during abiotic stress (cold, salt, drought). | Ma *et al.,* 2023; Marondedze *et al.,* 2019; Yan *et al.,* 2022 |
|  |  |  |  | Zm00001eb196240 | Auxin efflux carrier component | Regulates auxin transport and IAA homeostasis; modulates root growth under Al stress. | Allam *et al.,* 2025 |
|  | SChr4_169157503 | 4 | 169157503 | Zm00001eb189470 | Peptidase A1 domain–containing protein | Aspartic-type protease involved in protein degradation, ABA signaling, and drought stress adaptation. | Moloi & Ngara, 2023 |
|  |  |  |  | Zm00001eb189490 | Nuclear transcription factor Y subunit B-6 (NF-YB6) | Transcriptional regulator of root elongation and stress-responsive gene expression under abiotic stress. | Fu *et al.,* 2022; Kavi Kishor *et al*., 2023 |
|  | SChr7_137019441 | 7 | 137019441 | Zm00001eb316390 | Sodium/Hydrogen exchanger (Cation/H⁺ exchanger domain) | Regulates Na⁺/K⁺ balance and pH homeostasis during salt/alkaline stress via Na⁺ extrusion and K⁺ retention. | Jin *et al.,* 2022; Rahman *et al.,* 2022 |

**References:**

Allam, G., Sakariyahu, S.K., McDowell, T., Pitambar, T.A., Papadopoulos, Y., Bernards, M.A., Hannoufa, A., 2025. miR156 is a negative regulator of aluminium response in Medicago sativa. Plants 14, 958.

Fu, R., Wang, J., Zhou, M., Ren, X., Hua, J., Liang, M., 2022. Five NUCLEAR FACTOR-Y subunit B genes in rapeseed (Brassica napus) promote flowering and root elongation in Arabidopsis. Planta 256, 115.

Jin, T., An, J., Xu, H., Chen, J., Pan, L., Zhao, R., Wang, N., Gai, J., Li, Y., 2022. A soybean sodium/hydrogen exchanger GmNHX6 confers plant alkaline salt tolerance by regulating Na⁺/K⁺ homeostasis. Front. Plant Sci. 13, 938635.

Kavi Kishor, P.B., Ganie, S.A., Wani, S.H., Guddimalli, R., Karumanchi, A.R., Edupuganti, S., Naravula, J., Kumar, V., Polavarapu, R., Suravajhala, P., Penna, S., 2023. Nuclear Factor-Y (NF-Y): Developmental and stress-responsive roles in the plant lineage. J. Plant Growth Regul. 42, 2711–2735.

Ma, L., Tao, X., Wang, W., Jiao, J., Pu, Y., Yang, G., Liu, L., Fang, Y., Wu, J., Sun, W., 2023. Genome-wide identification of RNA recognition motif (RRM1) in Brassica rapa and functional analysis of RNA-binding protein (BrRBP) under low-temperature stress. BMC Plant Biol. 23, 621.

Marondedze, C., Thomas, L., Gehring, C., Lilley, K.S., 2019. Changes in the Arabidopsis RNA-binding proteome reveal novel stress response mechanisms. BMC Plant Biol. 19, 139.

Moloi, S.J., Ngara, R., 2023. The roles of plant proteases and protease inhibitors in drought response: A review. Front. Plant Sci. 14, 1165845.

Rahman, M.A., Woo, J.H., Lee, S.-H., Park, H.S., Kabir, A., Raza, A., El Sabagh, A., Lee, K.W., 2022. Regulation of Na⁺/H⁺ exchangers, Na⁺/K⁺ transporters, and lignin biosynthesis

Yan, Y., Gan, J., Tao, Y., Okita, T.W., Tian, L., 2022. RNA-binding proteins: The key modulator in stress granule formation and abiotic stress response. Front. Plant Sci. 13, 882596.
