# Supplementary material for: Genetic dissection of seedling root architecture under aluminium toxicity in tropical maize (Zea mays L.)
Source: Front Plant Sci. 2026 Feb 10;16:1722162. doi: 10.3389/fpls.2025.1722162 (PMC12929552; doi:10.3389/fpls.2025.1722162)
Supplement: Supplementary file 3 [file Table3.docx]

**Table S3 Putative candidate genes and molecular functions in the 65.4 kb region of linked SNPs for Number of Root Tips (NRT) under aluminium stress**

| **Trait** | **SNP** | **Chr** | **Position** | **Transcript ID** | **Protein** | **Role** | **References** |
| --- | --- | --- | --- | --- | --- | --- | --- |
| **NRT** | SChr8_136743030 | 8 | 136743030 | Zm00001eb355540 | DHAR-like1 (Dehydroascorbate reductase EC 1.8.5.1) | Recycles oxidized ascorbate to its reduced form, enhancing antioxidant capacity and tolerance to Al-induced oxidative damage. Overexpression improves stress tolerance without affecting Al accumulation. | Yin *et al.,* 2010 |
|  | SChr8_136742944 | 8 | 136742944 |  |  |  |  |
|  |  |  |  | Zm00001eb355550 | Glutathione S-transferase DHAR2 | GST27.2(Glutathione S-transferase)*,* a single-copy gene predominantly expressed in root tips and upregulated by Al exposure, likely contributing to detoxification and oxidative stress defense | Cançado *et al.,* 2005. |
|  |  |  |  | Zm00001eb355570 | Cupin type-1/Legumin-like protein | Functions as oxalate oxidase (OxO), generating ROS under Al stress. May contribute to both oxidative damage and defense via cell death in border cells to trap Al. | Dunwell *et al.,* 2008; Carrillo *et al.,* 2009 |
|  | SChr3_162691279 | 3 | 162691279 | Zm00001eb143280 | RRM domain-containing protein (GRP) | Glycine-rich RNA-binding protein (GRP) with RRM domain enhances stress tolerance via RNA processing and antioxidant regulation. Involved in ROS detoxification and osmolyte accumulation. | Ma *et al.,* 2021; Czolpinska & Rurek, 2018; Ortega-Amaro *et al.,* 2015; Kim *et al.,* 2007; Wang *et al.,* 2012; Yang *et al.,* 2014 |
|  | SChr3_162691266 | 3 | 162691266 |  |  |  |  |
|  | SChr3_162691487 | 3 | 162691487 |  |  |  |  |
|  |  |  |  | Zm00001eb143290 | CBS domain protein CBSX6 | Regulates abiotic stress responses via thioredoxin system; involved in fertility under heat stress; enhances drought tolerance. | Liu *et al.,* 2021; Ali *et al.,* 2021 |
|  | SChr2_220362889 | 2 | 220362889 | Zm00001eb158680 | Homeobox-leucine zipper protein ATHB-4 | HD-Zip II transcription factor regulating hormone-mediated (ABA, auxin, brassinosteroids) responses and shade avoidance. Involved in abiotic stress signaling. | Sorin *et al.,* 2009; Li *et al.,* 2022 |
|  | SChr1_160784456 | 1 | 160784456 | Zm00001eb029550 | Diacylglycerol kinase (DGK) | Converts DAG to phosphatidic acid (PA); involved in stress signaling including response to Al and other metals. | Escobar-Sepúlveda *et al.,* 2017 |
|  | SChr8_127618532 | 8 | 127618532 | Zm00001eb353510 | Kinesin-related protein 3 | Regulates ATP levels via mitochondrial VDAC interaction; responds to cold and salt stress. | Yang *et al.,* 2011; Jin *et al.,* 2025 |
|  | SChr8_127618207 | 8 | 127618207 |  |  |  |  |
|  |  |  |  | Zm00001eb353520 | Histidine-containing phosphotransfer protein | Negative regulator of drought tolerance; mutants show improved root traits, reduced ROS, and ABA-responsive gene upregulation. | Ha *et al.,* 2022 |
|  | SChr6_92882553 | 6 | 92882553 | Zm00001eb272150 | bHLH93 transcription factor | Enhances osmotic stress tolerance via ABA signaling, stomatal closure, ROS balance, and osmolyte accumulation. | Yang *et al.,* 2016; Zhao *et al.,* 2023 |
|  |  |  |  | Zm00001eb272160 | Transducin/WD40 repeat-like superfamily protein | Acts as scaffolding protein; regulates development, stress signaling, and ribosome biogenesis. | Gachomo *et al.,* 2014; Sharma & Pandey, 2016 |
|  |  |  |  | Zm00001eb272170 | DNL-type domain-containing protein | Enhances drought/salt tolerance through ROS scavenging; important for abiotic stress adaptation. | Li *et al.,* 2023 |
|  | SChr3_24883074 | 3 | 24883074 | Zm00001eb125730 | Heavy metal detoxification protein | MATE/ABC transporters involved in detoxification of Al, Cd, Hg. Enhance tolerance by sequestration and transport. | Yokosho *et al.,* 2014; Paape *et al.,* 2022 |

**References:**

Ali, F., Li, Y., Li, F., Wang, Z., 2021. Genome-wide characterization and expression analysis of cystathionine β-synthase genes in plant development and abiotic stresses of cotton (Gossypium spp.). Int. J. Biol. Macromol. 193, 823–837.

Cañçado, G.M.A., De Rosa Jr, V.E., Fernandez, J.H., Maron, L.G., Jorge, R.A., Menossi, M., 2005. Glutathione S-transferase and aluminium toxicity in maize. Funct. Plant Biol. 32, 1045–1055.

Carrillo, M.G.C., Goodwin, P.H., Leach, J.E., et al., 2009. Phylogenomic relationships of rice oxalate oxidases to the cupin superfamily and their association with disease resistance QTL. Rice 2, 67–79.

Czolpinska, M., Rurek, M., 2018. Plant glycine-rich proteins in stress response: An emerging, still prospective story. Front. Plant Sci. 9, 302.

Dunwell, J., Gibbings, J.G., Mahmood, T., Saqlan Naqvi, S.M., 2008. Germin and germin-like proteins: evolution, structure, and function. Crit. Rev. Plant Sci. 27, 342–375.

Escobar-Sepulveda, H.F., Trejo-Téllez, L.I., Pérez-Rodríguez, P., Hidalgo-Contreras, J.V., Gomez-Merino, F.C., 2017. Diacylglycerol kinases are widespread in higher plants and display inducible gene expression in response to beneficial elements, metal, and metalloid ions. Front. Plant Sci. 8, 129.

Gachomo, E.W., Jimenez-Lopez, J.C., Baptiste, L.J., Kotchoni, S.O., 2014. GIGANTUS1 (GTS1), a member of transducin/WD40 protein superfamily, controls seed germination, growth and biomass accumulation through ribosome-biogenesis protein interactions in Arabidopsis thaliana. BMC Plant Biol. 14, 37.

Ha, C.V., Mostofa, M.G., Nguyen, K.H., Tran, C.D., Watanabe, Y., Li, W., Osakabe, Y., Sato, M., Toyooka, K., Tanaka, M., et al., 2022. The histidine phosphotransfer AHP4 plays a negative role in Arabidopsis plant response to drought. Plant J. 111, 1732–1752.

Jin, T., Zhang, K., Zhang, X., Wu, C., Long, W., 2025. Genome-wide identification of the kinesin gene family in soybean and its response to salt stress. Agronomy 15, 275.

Kim, J.Y., Park, S.J., Jang, B., Jung, C.H., Ahn, S.J., Goh, C.H., et al., 2007. Functional characterization of a glycine-rich RNA-binding protein2 in Arabidopsis thaliana under abiotic stress conditions. Plant J. 50, 439–451.

Li, K., Zhai, L., Fu, S., Wu, T., Zhang, X., Xu, X., Han, Z., Wang, Y., 2023. Genome-wide analysis of the MdZR gene family revealed MdZR2.2-induced salt and drought stress tolerance in apple rootstock. Plant Sci. 334, 111755.

Li, Y., Yang, Z., Zhang, Y., Guo, J., Liu, L., Wang, C., Wang, B., Han, G., 2022. The roles of HD-ZIP proteins in plant abiotic stress tolerance. Front. Plant Sci. 13, 1027071.

Liu, H., Sun, Z., Hu, L., Li, C., Wang, X., Yue, Z., Han, Y., Yang, G., Ma, K., Yin, G., 2021. Comparative transcriptome analysis of male sterile anthers induced by high temperature in wheat (Triticum aestivum L.). Front. Plant Sci. 12, 727966.

Ma, L., Cheng, K., Li, J., Deng, Z., Zhang, C., Zhu, H., 2021. Roles of plant glycine-rich RNA-binding proteins in development and stress responses. Int. J. Mol. Sci. 22, 5849.

Ortega-Amaro, M.A., Rodriguez-Hernández, A.A., Rodríguez-Kessler, M., Hernandez-Lucero, E., Rosales-Mendoza, S., Ibanez-Salazar, A., Delgado-Sánchez, P., Jimenez-Bremont, J.F., 2015. Overexpression of AtGRDP2, a novel glycine-rich domain protein, accelerates plant growth and improves stress tolerance. Front. Plant Sci. 5, 782.

Paape, T., Heiniger, B., Santo Domingo, M., Clear, M.R., Lucas, M.M., Pueyo, J.J., 2022. Genome-wide association study reveals complex genetic architecture of cadmium and mercury accumulation and tolerance traits in Medicago truncatula. Front. Plant Sci. 12, 806949.

Sharma, M., Pandey, G.K., 2016. Expansion and function of repeat domain proteins during stress and development in plants. Front. Plant Sci. 6, 1218.

Sorin, C., Salla-Martret, M., Bou-Torrent, J., Roig-Villanova, I., Martínez-García, J.F., 2009. ATHB4, a regulator of shade avoidance, modulates hormone response in Arabidopsis seedlings. Plant J. 59, 266–277.

Wang, C., Zhang, D.W., Wang, Y.C., Zheng, L., Yang, C.P., 2012. A glycine-rich RNA-binding protein can mediate physiological responses in transgenic plants under salt stress. Mol. Biol. Rep. 39, 1047–1053.

Yang, D.H., Kwak, K.J., Kim, M.K., Park, S.J., Yang, K.-Y., Kang, H., 2014. Expression of Arabidopsis glycine-rich RNA-binding protein AtGRP2 or AtGRP7 improves grain yield of rice (Oryza sativa) under drought stress conditions. Plant Sci. 214, 106–112.

Yang, T., Yao, S., Hao, L., Zhao, Y., Lu, W., Xiao, K., 2016. Wheat bHLH-type transcription factor gene TabHLH1 is crucial in mediating osmotic stress tolerance through modulating largely the ABA-associated pathway. Plant Cell Rep. 35, 2309–2323.

Yang, X.Y., Chen, Z.W., Xu, T., Qu, Z., Pan, X.D., Qin, X.H., Ren, D.T., Liu, G.Q., 2011. Arabidopsis kinesin KP1 specifically interacts with VDAC3, a mitochondrial protein, and regulates respiration during seed germination at low temperature. Plant Cell 23, 1093–1106.

Yin, L., Wang, S., Eltayeb, A.E., Uddin, M.I., Yamamoto, Y., Tsuji, W., Takeuchi, Y., Tanaka, K., 2010. Overexpression of dehydroascorbate reductase, but not monodehydroascorbate reductase, confers tolerance to aluminium stress in transgenic tobacco. Planta 231, 609–621.

Yokosho, K., Yamaji, N., Ma, J.F., 2014. Global transcriptome analysis of Al-induced genes in an Al-accumulating species, common buckwheat (Fagopyrum esculentum Moench). Plant Cell Physiol. 55, 2077–2091.

Zhao, L., Bi, W., Jia, Y., Shi, J., Chi, Y., Yu, M., Wang, C., 2023. Genome-wide characterization of bHLH family genes and expression analysis in response to osmotic stress in Betula platyphylla. Plants 12, 3687.
