# Supplementary material for: Genetic dissection of seedling root architecture under aluminium toxicity in tropical maize (Zea mays L.)
Source: Front Plant Sci. 2026 Feb 10;16:1722162. doi: 10.3389/fpls.2025.1722162 (PMC12929552; doi:10.3389/fpls.2025.1722162)
Supplement: Supplementary file 4 [file Table4.docx]

**Table S4 Putative candidate genes and molecular functions in the 65.4 kb region of linked SNPs for Root Volume (RV) under aluminium stress**

| **Trait** | **SNP** | **Chro** | **Position** | **Transcript ID** | **Protein** | **Role** | **Reference(s)** |
| --- | --- | --- | --- | --- | --- | --- | --- |
| **RV** | SChr6_172447002 | 6 | 172447002 | Zm00001eb293920 | Non-structural maintenance of chromosomes element 4 | Regulates root cell proliferation and stem cell maintenance via cell cycle and cytokinin signaling; involved in DNA repair. | Huang *et al.,* 2009; Xu *et al.,* 2013;  Li *et al.,* 2017; Watanabe *et al.,* 2009 |
|  |  |  |  | Zm00001eb293930 | F-box domain-containing protein | Enhances stress tolerance (drought, salt, ABA); F-box proteins are key regulators in stress signaling pathways. | Song *et al.,* 2015;  Zhou *et al.,* 2014; Xu *et al.,* 2014 |
|  | SChr6_161539730 | 6 | 161539730 | Zm00001eb289200 | Serine/threonine-protein kinase SRK2A | Key ABA-activated kinase (SnRK2.6/OST1); regulates seed dormancy and stress responses. | Nakashima *et al.,* 2009;  Li *et al.,* 2000; Mustilli *et al.,* 2002; Yoshida *et al.,* 2002; Zentella *et al.,* 2002 |
|  | SChr7_102943539 | 7 | 102943539 | Zm00001eb310980 | THO complex subunit 6 | Regulates mRNA export, small RNA biogenesis, and splicing—critical for Al stress tolerance in Arabidopsis. | Zhu *et al.,* 2021; Guo *et al.,* 2020;  Pan *et al.,* 2012; Tao *et al.,* 2016;  Xu *et al.,* 2015; Jauvion *et al.,* 2010;  Khan *et al.,* 2020; Yelina *et al.,* 2010 |
|  | SChr8_127618532 | 8 | 127618532 | Zm00001eb353510 | Kinesin-related protein3 | Involved in mitochondrial function during germination; contributes to salt tolerance in soybean. | Yang *et al.,* 2011; Jin *et al.,* 2025 |
|  |  |  |  | Zm00001eb353520 | Histidine-containing phosphotransfer protein | AHP4 is a negative regulator of drought tolerance; its loss improves root and ABA-responsive traits. | Ha *et al.,* 2022 |

**References:**

Guo, J., Zhang, Y., Gao, H., Li, S., Wang, Z.Y., Huang, C.F., 2020. Mutation of HPR1 encoding a component of the THO/TREX complex reduces STOP1 accumulation and aluminium resistance in Arabidopsis thaliana. New Phytol. 228, 179–193.

Ha, C.V., Mostofa, M.G., Nguyen, K.H., Tran, C.D., Watanabe, Y., Li, W., Osakabe, Y., Sato, M., Toyooka, K., Tanaka, M., et al., 2022. The histidine phosphotransfer AHP4 plays a negative role in Arabidopsis plant response to drought. Plant J. 111, 1732–1752.

Huang, L., Yang, S., Zhang, S., Liu, M., Lai, J., Qi, Y., Shi, S., Wang, J., Wang, Y., Xie, Q., 2009. The Arabidopsis SUMO E3 ligase AtMMS21, a homologue of NSE2/MMS21, regulates cell proliferation in the root. Plant J. 60, 666–678.

Jauvion, V., Elmayan, T., Vaucheret, H., 2010. The conserved RNA trafficking proteins HPR1 and TEX1 are involved in the production of endogenous and exogenous small interfering RNA in Arabidopsis. Plant Cell 22, 2697–2709.

Jin, T., Zhang, K., Zhang, X., Wu, C., Long, W., 2025. Genome-wide identification of the kinesin gene family in soybean and its response to salt stress. Agronomy 15, 275.

Khan, G.A., Deforges, J., Reis, R.S., Hsieh, Y.F., Montpetit, J., Antosz, W., Santuari, L., Hardtke, C.S., Grasser, K.D., Poirier, Y., 2020. The transcription and export complex THO/TREX contributes to transcription termination in plants. PLoS Genet. 16, e1008732.

Li, G., Zou, W., Jian, L., Qian, J., Deng, Y., Zhao, J., 2017. Non-SMC elements 1 and 3 are required for early embryo and seedling development in Arabidopsis. J. Exp. Bot. 68, 1039–1054.

Li, J., Wang, X.Q., Watson, M.B., Assmann, S.M., 2000. Regulation of abscisic acid-induced stomatal closure and anion channels by guard cell AAPK kinase. Science 287, 300–303.

Mustilli, A.C., Merlot, S., Vavasseur, A., Fenzi, F., Giraudat, J., 2002. Arabidopsis OST1 protein kinase mediates the regulation of stomatal aperture by abscisic acid and acts upstream of reactive oxygen species production. Plant Cell 14, 3089–3099.

Nakashima, K., Fujita, Y., Kanamori, N., Katagiri, T., Umezawa, T., Kidokoro, S., Maruyama, K., Yoshida, T., Ishiyama, K., Kobayashi, M., Shinozaki, K., Yamaguchi-Shinozaki, K., 2009. Three Arabidopsis SnRK2 protein kinases, SRK2D/SnRK2.2, SRK2E/SnRK2.6/OST1 and SRK2I/SnRK2.3, involved in ABA signaling are essential for the control of seed development and dormancy. Plant Cell Physiol. 50, 1345–1363.

Pan, H., Liu, S., Tang, D., 2012. HPR1, a component of the THO/TREX complex, plays an important role in disease resistance and senescence in Arabidopsis. Plant J. 69, 831–843.

Song, J.B., Wang, Y.X., Li, H.B., Li, B.W., Zhou, Z.S., Gao, S., Yang, Z.M., 2015. The F-box family genes as key elements in response to salt, heavy metal, and drought stresses in Medicago truncatula. Funct. Integr. Genomics 15, 495–507.

Tao, S., Zhang, Y., Wang, X., Xu, L., Fang, X., Lu, Z.J., Liu, D., 2016. The THO/TREX complex active in miRNA biogenesis negatively regulates root-associated acid phosphatase activity induced by phosphate starvation. Plant Physiol. 171, 2841–2853.

Watanabe, K., Pacher, M., Dukowic, S., Schubert, V., Puchta, H., Schubert, I., 2009. The STRUCTURAL MAINTENANCE OF CHROMOSOMES 5/6 complex promotes sister chromatid alignment and homologous recombination after DNA damage in Arabidopsis thaliana. Plant Cell 21, 2688–2699.

Xu, C., Zhou, X., Wen, C.K., 2015. HYPER RECOMBINATION1 of the THO/TREX complex plays a role in controlling transcription of the REVERSION-TO-ETHYLENE SENSITIVITY1 gene in Arabidopsis. PLoS Genet. 11, e1004956.

Xu, G., Cui, Y., Wang, M., Li, M., Yin, X., Xia, X., 2014. OsMsr9, a novel putative rice F-box containing protein, confers enhanced salt tolerance in transgenic rice and Arabidopsis. Mol. Breed. 34, 1055–1064.

Xu, P., Yuan, D., Liu, M., Li, C., Liu, Y., Zhang, S., Yao, N., Yang, C., 2013. AtMMS21, an SMC5/6 complex subunit, is involved in stem cell niche maintenance and DNA damage responses in Arabidopsis roots. Plant Physiol. 161, 1755–1768.

Yang, X.Y., Chen, Z.W., Xu, T., Qu, Z., Pan, X.D., Qin, X.H., Ren, D.T., Liu, G.Q., 2011. Arabidopsis kinesin KP1 specifically interacts with VDAC3, a mitochondrial protein, and regulates respiration during seed germination at low temperature. Plant Cell 23, 1093–1106.

Yelina, N.E., Smith, L.M., Jones, A.M., Patel, K., Kelly, K.A., Baulcombe, D.C., 2010. Putative Arabidopsis THO/TREX mRNA export complex is involved in transgene and endogenous siRNA biosynthesis. Proc. Natl. Acad. Sci. U.S.A. 107, 13948–13953.

Yoshida, R., Hobo, T., Ichimura, K., Mizoguchi, T., Takahashi, F., Aronso, J., Ecker, J.R., Shinozaki, K., 2002. ABA-activated SnRK2 protein kinase is required for dehydration stress signaling in Arabidopsis. Plant Cell Physiol. 43, 1473–1483.

Zentella, R., Yamauchi, D., Ho, T.H., 2002. Molecular dissection of the gibberellin/abscisic acid signaling pathways by transiently expressed RNA interference in barley aleurone cells. Plant Cell 14, 2289–2301.

Zhou, S., Sun, X., Yin, S., Kong, X., Zhou, S., Xu, Y., Luo, Y., Wang, W., 2014. The role of the F-box gene TaFBA1 from wheat (Triticum aestivum L.) in drought tolerance. Plant Physiol. Biochem. 84, 213–223.

Zhu, Y.F., Guo, J., Zhang, Y., Huang, C.F., 2021. The THO/TREX complex component RAE2/TEX1 is involved in the regulation of aluminum resistance and low phosphate response in Arabidopsis. Front. Plant Sci. 12, 698443.
