# Supplementary material for: Genetic dissection of seedling root architecture under aluminium toxicity in tropical maize (Zea mays L.)
Source: Front Plant Sci. 2026 Feb 10;16:1722162. doi: 10.3389/fpls.2025.1722162 (PMC12929552; doi:10.3389/fpls.2025.1722162)
Supplement: Supplementary file 5 [file Table5.docx]

**Table S5 Putative candidate genes and molecular functions in the 65.4 kb region of linked SNPs for Average Root Diameter (AD) under aluminium stress**

| **Trait** | **SNP** | **Chro** | **Position** | **Transcript ID** | **Protein** | **Role** | **Reference** |
| --- | --- | --- | --- | --- | --- | --- | --- |
| **AD** | SChr3_147147013 | 3 | 147147013 | Zm00001eb139930 | mRNA cap-binding protein | Modulates early ABA signaling; enhances drought tolerance and stomatal closure. | Hugouvieux *et al.,* 2001;  Daszkowska-Golec *et al.,* 2017 |
|  | SChr3_147146931 | 3 | 147146931 |  |  |  |  |
|  |  |  |  | Zm00001eb139940 | ATP-dependent DNA helicase | Enhances stress tolerance via DNA/RNA metabolism; improves antioxidant system. | Tuteja *et al.,* 2014; Anand & Pandi, 2021; Sahoo *et al.,* 2022a |
|  | SChr3_11527008 | 3 | 11527008 | Zm00001eb122720 | NADH-cytochrome b5 reductase | Boosts ATP via β-oxidation; enhances drought and salinity stress tolerance. | Gao *et al.,* 2025 |
|  | SChr3_11526962 | 3 | 11526962 |  |  |  |  |
|  |  |  |  | Zm00001eb122730 | MAPK kinase substrate protein | Mediates MAPK signaling; activates stress-responsive gene expression. | Dóczi, 2011 |
|  | SChr4_210113698 | 4 | 210113698 | Zm00001eb200780 | Fe2OG dioxygenase domain-containing protein | Catalyzes flavonoid biosynthesis; aids stress adaptation via metabolic shifts. | Wang *et al.,* 2021 |
|  |  |  |  | Zm00001eb200790 | Aspartyl protease family protein | Involved in pathogen defense; mediates stress and developmental responses. | Figueiredo *et al.,* 2021 |
|  | SChr8_131335005 | 8 | 131335005 | Zm00001eb354260 | Glutathione transferase; GST N-terminal domain protein | Detoxifies ROS and metals; involved in aluminium and oxidative stress defense. | Dmitriev *et al.,* 2016; Vijayakumar *et al.,* 2016;  Kumar & Trivedi, 2018 |
|  | SChr8_131335018 | 8 | 131335018 |  |  |  |  |
|  |  |  |  | Zm00001eb354270 | Thioredoxin domain protein; TRX F2 chloroplastic | Regulates redox state, photosynthesis, ABA sensitivity, and oxidative stress tolerance. | Djoumad *et al.,* 2018;  Serrato *et al.,* 2013 |

**References:**

Anand, A., Pandi, G., 2021. Noncoding RNA: An insight into the chloroplast and mitochondrial gene expressions. Life 11, 49–69.

Daszkowska-Golec, A., Szarejko, I., Maluszynski, M., 2017. Mutation in HvCBP20 (cap binding protein 20) adapts barley to drought stress at phenotypic and transcriptomic levels. Front. Plant Sci. 8, 942.

Djoumad, A., Villette, S., Isayenka, I., Beaudoin, N., 2018. Involvement of type-f thioredoxins during germination and early seedling development and in response to oxidative stress in Arabidopsis thaliana. Botany 96, 471–484.

Dmitriev, A.A., Krasnov, G.S., Rozhmina, T.A., Kishlyan, N.V., Zyablitsin, A.V., Sadritdinova, A.F., Snezhkina, A.V., Fedorova, M.S., Yurkevich, O.Y., Muravenko, O.V., Bolsheva, N.L., Kudryavtseva, A.V., Melnikova, N.V., 2016. Glutathione S-transferases and UDP-glycosyltransferases are involved in response to aluminium stress in flax. Front. Plant Sci. 7, 1920.

Doczi, R., 2011. Mitogen-activated protein (MAP) kinase signalling in plant environmental stress responses. Acta Agron. Hung. 59, 285–290.

Figueiredo, L., Santos, R.B., Figueiredo, A., 2021. Defense and offense strategies: The role of aspartic proteases in plant–pathogen interactions. Biology 10, 75.

Gao, Y., Chen, A., Zhu, D., Zhou, M., Huang, H., Pan, R., Wang, X., Li, L., Shen, J., 2025. Mitochondrial energy homeostasis and membrane interaction regulate the rapid growth of moso bamboo. Plant Cell Environ. 48, 5874–5896.

Hugouvieux, V., Kwak, J.M., Schroeder, J.I., 2001. An mRNA cap-binding protein, ABH1, modulates early abscisic acid signal transduction in Arabidopsis. Cell 106, 477–487.

Kumar, S., Trivedi, P.K., 2018. Glutathione S-transferases: Role in combating abiotic stresses including arsenic detoxification in plants. Front. Plant Sci. 9, 751.

Sahoo, R.K., Chandan, R.K., Swain, D.M., Tuteja, N., Jha, G., 2022a. Heterologous overexpression of PDH45 gene of pea provides tolerance against sheath blight disease and drought stress in rice. Plant Physiol. Biochem. 186, 242–251.

Serrato, A.J., Fernández-Trijueque, J., Barajas-López, J.-d.-D., Chueca, A., Sahrawy, M., 2013. Plastid thioredoxins: A “one-for-all” redox-signaling system in plants. Front. Plant Sci. 4, 463.

Tuteja, N., Tarique, M., Tuteja, R., 2014. Rice SUV3 is a bidirectional helicase that binds both DNA and RNA. BMC Plant Biol. 14, 283.

Vijayakumar, H., Thamilarasan, S.K., Shanmugam, A., Natarajan, S., Jung, H.J., Park, J.I., Kim, H., Chung, M.Y., Nou, I.S., 2016. Glutathione transferases superfamily: Cold-inducible expression of distinct GST genes in Brassica oleracea. Int. J. Mol. Sci. 17, 1211.

Wang, Y., Shi, Y., Li, K., Yang, D., Liu, N., Zhang, L., Zhao, L., Zhang, X., Liu, Y., Gao, L., Xia, T., Wang, P., 2021. Roles of the 2-oxoglutarate-dependent dioxygenase superfamily in the flavonoid pathway: A review of the functional diversity of F3H, FNS I, FLS, and LDOX/ANS. Molecules 26, 6745.
