# Supplementary material for: Genetic dissection of seedling root architecture under aluminium toxicity in tropical maize (Zea mays L.)
Source: Front Plant Sci. 2026 Feb 10;16:1722162. doi: 10.3389/fpls.2025.1722162 (PMC12929552; doi:10.3389/fpls.2025.1722162)
Supplement: Supplementary file 6 [file Table6.docx]

**Table S6 Putative candidate genes and molecular functions in the 65.4 kb region of linked SNPs for Root Surface Area (RSA) under aluminium stress**

| **Trait** | **SNP** | **Chro** | **Position** | **Transcript ID** | **Protein** | **Role** | **Reference(s)** |
| --- | --- | --- | --- | --- | --- | --- | --- |
| **RSA** | SChr8_127618532 | 8 | 127618532 | Zm00001eb353510 | Kinesin-related protein 3 | Regulates mitochondrial respiration and ATP via VDAC3 interaction during stress | Yang *et al.,* 2011;  Jin *et al.,* 2025 |
|  | SChr8_127618207 | 8 | 127618207 |  |  |  |  |
|  |  |  |  | Zm00001eb353520 | Histidine-containing phosphotransfer protein | AHP4 negatively regulates drought tolerance via ABA pathway, ROS reduction, and root trait improvement | Ha *et al.,* 2022 |
|  | SChr7_102943539 | 7 | 102943539 | Zm00001eb310980 | THO complex subunit 6 | Involved in mRNA export, splicing, and sRNA biogenesis for Al resistance | Zhu *et al.,* 2021  Guo *et al.,* 2020  Pan *et al.,* 2012  Tao *et al.,* 2016  Xu *et al.,* 2015  Jauvion *et al.,* 2010  Khan *et al.,* 2020  Yelina *et al.,* 2010 |

**References:**

Gao, H., Ye, S., Wu, J., Wang, L., Wang, R., Lei, W., Meng, L., Yuan, F., Zhou, Q., Cui, C., 2020. Genome-wide association analysis of aluminium tolerance-related traits in rapeseed (Brassica napus L.) during germination. Euphytica 216, 71.

Ha, C.V., Mostofa, M.G., Nguyen, K.H., Tran, C.D., Watanabe, Y., Li, W., Osakabe, Y., Sato, M., Toyooka, K., Tanaka, M., et al., 2022. The histidine phosphotransfer AHP4 plays a negative role in Arabidopsis plant response to drought. Plant J. 111, 1732–1752.

Jauvion, V., Elmayan, T., Vaucheret, H., 2010. The conserved RNA trafficking proteins HPR1 and TEX1 are involved in the production of endogenous and exogenous small interfering RNA in Arabidopsis. Plant Cell 22, 2697–2709.

Jin, T., Zhang, K., Zhang, X., Wu, C., Long, W., 2025. Genome-wide identification of the kinesin gene family in soybean and its response to salt stress. Agronomy 15, 275.

Khan, G.A., Deforges, J., Reis, R.S., Hsieh, Y.F., Montpetit, J., Antosz, W., Santuari, L., Hardtke, C.S., Grasser, K.D., Poirier, Y., 2020. The transcription and export complex THO/TREX contributes to transcription termination in plants. PLoS Genet. 16, e1008732.

Pan, H., Liu, S., Tang, D., 2012. HPR1, a component of the THO/TREX complex, plays an important role in disease resistance and senescence in Arabidopsis. Plant J. 69, 831–843.

Tao, S., Zhang, Y., Wang, X., Xu, L., Fang, X., Lu, Z.J., Liu, D., 2016. The THO/TREX complex active in miRNA biogenesis negatively regulates root-associated acid phosphatase activity induced by phosphate starvation. Plant Physiol. 171, 2841–2853.

Xu, C., Zhou, X., Wen, C.K., 2015. HYPER RECOMBINATION1 of the THO/TREX complex plays a role in controlling transcription of the REVERSION-TO-ETHYLENE SENSITIVITY1 gene in Arabidopsis. PLoS Genet. 11, e1004956.

Yang, X.Y., Chen, Z.W., Xu, T., Qu, Z., Pan, X.D., Qin, X.H., Ren, D.T., Liu, G.Q., 2011. Arabidopsis kinesin KP1 specifically interacts with VDAC3, a mitochondrial protein, and regulates respiration during seed germination at low temperature. Plant Cell 23, 1093–1106.

Yelina, N.E., Smith, L.M., Jones, A.M., Patel, K., Kelly, K.A., Baulcombe, D.C., 2010. Putative Arabidopsis THO/TREX mRNA export complex is involved in transgene and endogenous siRNA biosynthesis. Proc. Natl. Acad. Sci. U.S.A. 107, 13948–13953.

Zhu, Y.F., Guo, J., Zhang, Y., Huang, C.F., 2021. The THO/TREX complex component RAE2/TEX1 is involved in the regulation of aluminum resistance and low phosphate response in Arabidopsis. Front. Plant Sci. 12, 698443.
